# *Escherichia coli* DnaE Polymerase Couples Pyrophosphatase Activity to DNA Replication

**DOI:** 10.1371/journal.pone.0152915

**Published:** 2016-04-06

**Authors:** Fabio Lapenta, Alejandro Montón Silva, Renato Brandimarti, Massimiliano Lanzi, Fabio Lino Gratani, Perceval Vellosillo Gonzalez, Sofia Perticarari, Alejandro Hochkoeppler

**Affiliations:** 1 Department of Pharmacy and Biotechnology, University of Bologna, Viale Risorgimento 4, 40136, Bologna, Italy; 2 Department of Industrial Chemistry, University of Bologna, Viale Risorgimento 4, 40136, Bologna, Italy; 3 CSGI, University of Firenze, Via della Lastruccia 3, 50019, Sesto Fiorentino, FI, Italy; Institute of Molecular Genetics IMG-CNR, ITALY

## Abstract

DNA Polymerases generate pyrophosphate every time they catalyze a step of DNA elongation. This elongation reaction is generally believed as thermodynamically favoured by the hydrolysis of pyrophosphate, catalyzed by inorganic pyrophosphatases. However, the specific action of inorganic pyrophosphatases coupled to DNA replication *in vivo* was never demonstrated. Here we show that the Polymerase-Histidinol-Phosphatase (PHP) domain of *Escherichia coli* DNA Polymerase III α subunit features pyrophosphatase activity. We also show that this activity is inhibited by fluoride, as commonly observed for inorganic pyrophosphatases, and we identified 3 amino acids of the PHP active site. Remarkably, *E*. *coli* cells expressing variants of these catalytic residues of α subunit feature aberrant phenotypes, poor viability, and are subject to high mutation frequencies. Our findings indicate that DNA Polymerases can couple DNA elongation and pyrophosphate hydrolysis, providing a mechanism for the control of DNA extension rate, and suggest a promising target for novel antibiotics.

## Introduction

DNA Polymerases (DNA Pols) catalyze the extension of primers annealed to DNA template strands [1,2]. These enzymes promote the nucleophilic attack by the 3’-OH of the primer to the α-phosphate of an incoming deoxynucleotide triphosphate (dNTP), releasing pyrophosphate [3]. DNA Pols also catalyze the reverse reaction, denoted as pyrophosphorolysis, consisting in the shortening of DNA and the release of a dNTP [4]. Therefore, DNA replication is favoured by the concomitant hydrolysis of pyrophosphate, the ΔG of which is strongly negative [5]. This was early recognized [4], and inorganic pyrophosphatases (PPases) were claimed to be responsible for pushing the equilibrium of the reaction towards DNA extension [1]. However, no evidence was provided supporting the idea that the action of inorganic PPase(s) is coupled to DNA replication *in vivo*.

DNA Pols feature a conserved molecular architecture, consisting of 3 main domains, i.e. thumb, palm and fingers. The overall shape of these enzymes resembles an open right hand, with the catalytic site universally located in the palm. Quite recently, it was reported that some DNA Pols contain an additional domain, i.e. the Polymerase-Histidinol-Phosphatase (PHP) domain [6], the presence of which is frequently observed in the enzymes responsible for genome replication [7]. In *E*. *coli*, the organism whose DNA Pols are the best characterized, the only DNA replicase containing a PHP domain is the α subunit of DNA Pol III, the assembly of which requires 10 different subunits [2]. In addition, it was demonstrated that: i) *E*. *coli* is able to express 5 different DNA Pols (I-V) [8–13]; ii) DNA Pol III is essential for genome replication [14,15]; iii) DNA Pols II, IV, and V are dispensable [16–18]; iv) the 5’-3’ exonuclease domain of Pol I is essential to remove the primers generated during the replication of *E*. *coli* chromosome [19]; v) the Polymerase domain of Pol I is dispensable [20]. Therefore, the presence of PHP in the only essential DNA Pol suggests a functional role for this domain, although its presence could be due to structural reasons [21]. Favouring the functional role, Aravind and Koonin suggested that the PHP domain could feature pyrophosphatase activity [6]. Quite recently, the tertiary structure of a truncated form of *E*. *coli* DNA Pol III α subunit was determined, and, intriguingly, a phosphate ion associated to the PHP domain was detected [22]. The tertiary structure of PHP consists of a distorted α/β barrel, containing 6 but one parallel β strands. Remarkably, the phosphate ion bound to PHP is located at the C-terminal side of the β-strands, where it would be expected the PHP active site.

Here we show that the PHP domain of *E*. *coli* DNA Pol III α subunit features fluoride-sensitive pyrophosphatase activity. We also identified the PHP active site and, using purified site-specific variants of α subunit, we revealed a strong coupling between the rates of DNA elongation and pyrophosphatase activities. Considering the defective phenotypes linked to these variants of α subunit, we propose that the pyrophosphatase activity of DNA replicases represents a regulatory point of proper genome replication.

## Materials and Methods

### Bacterial Cultures

*Escherichia coli* TOP10 (genotype: F^-^ *mcrA* Δ(*mrr-hsdRMS-mcrBC*) ϕ80*lacZΔM15* Δ*lacX74 recA1 araD139* Δ(*ara-leu*)*7697 galU galK rpsL endA1 nupG*) was used to overexpress all the proteins considered. Full-length wt, H12A, and D19A α-subunits, along with the wt τ_3_α_3_ε_3_θ_3_ and the τ_3_α(D201A)_3_ε_3_θ_3_ complex were overexpressed as previously described [23,24]. To overexpress the PHP domain, *E*. *coli* TOP10 was transformed with the pBAD-α187 vector [23]. The transformants accordingly obtained were pre-cultured at 37°C for 15 h under shaking (180 rpm), using LB medium supplemented with ampicillin at 0.1 mg/mL. The pre-cultures were diluted (1:500) in fresh LB-ampicillin medium and grown, under the same conditions, for 5 h. Cultures were then induced to overexpress the PHP domain by the addition of 1 mM arabinose, and the induction was maintained for 15 h. Finally, cells were collected by centrifugation (5,000xg, 20 min, 4°C), and stored at -20°C. Using this overexpression procedure, inclusion bodies containing the PHP domain were produced.

### Construction of pGOOD-τ_γless_-ε-θ

The *dna*X-γless gene was synthesized by Entelechon GmbH (Bad Abbach, Germany). To avoid overexpression of the γ subunit, the sequence responsible for the translational frameshift yielding γ was mutated [25–27], i.e. the wild type codons 429–431 were changed to AAG-AAA-AGC. The pGOOD-τ_γless_-ε-θ vector, previously described [24], was used to co-transform *E*. *coli* TOP10 with the pBAD-α or with the pBAD-αD201A plasmid. The co-transformants accordingly obtained were then utilized to overexpress the wt τ_3_α_3_ε_3_θ_3_ and the τ_3_α(D201A)_3_ε_3_θ_3_ complex.

### Protein Purification

Full-length wt, H12A, and D19A α subunits, the τ_3_α_3_ε_3_θ_3_ and the τ_3_α(D201A)_3_ε_3_θ_3_ complex were purified as previously described [24].

To purify the PHP domain, frozen cells containing the overexpressed protein were gently thawed, and resuspended in 1/10 of the original culture volume using 50 mM Tris-HCl (pH 8), 50 mM NaCl, 1 mM EDTA (buffer A). The cells suspension was homogenized with a cold potter, and 1 mM phenyl-methyl-sulfonyl fluoride (PMSF) was added. Inclusion bodies were extracted by 3 sonication cycles, using a Misonix 3000 sonifier (Farmingdale, NY, USA) at an output level of 6 W. Each sonication cycle consisted of 15 s of pulse, followed by a 15 s cooling interval for a total time of 2 min. Inclusion bodies were then recovered by centrifugation (10,000xg, 20 min), and resuspended in buffer A containing 0.1% (v/v) Triton-X-100 (buffer B). The suspension was centrifuged, and the washing step with buffer B was repeated twice. Finally, the pellet (1.5 g) containing the inclusion bodies was solubilized in 300 mL of 50 mM Tris-HCl (pH 8), 50 mM NaCl, 1 mM EDTA, 5 mM DTT, 6 M urea. After incubation for 2 h at room temperature under stirring, the solubilized pellet was diluted (1:2) with buffer A supplemented with 5 mM DTT and 20% (v/v) glycerol (buffer C). Finally, the sample was concentrated to 100 mL with an Amicon ultrafiltration cell equipped with a YM30 membrane, and dialyzed (3 cycles) against buffer C. After dialysis, the solution was centrifuged (10,000xg, 20 min at 4°C), the pellet was discarded and the supernatant, containing 1.4 mg of proteins/mL, was concentrated to 35 mL. The sample was again centrifuged (10,000xg, 20 min at 4°C), and the supernatant, featuring 1.6 mg of proteins/mL, was loaded onto a Q-Sepharose FF column (1.6x25 cm, GE Healthcare, Piscataway, USA) previously equilibrated with buffer C. After sample loading, the column was washed with 5 column volumes of buffer C, and elution was performed applying a linear NaCl gradient (from 50 mM to 1 M). PHP domain was eluted at about 0.45 M NaCl. The best fractions, according to SDS-PAGE analysis, were pooled, concentrated, and loaded onto a Superdex 200 column (GE Healthcare, 1.6x70 cm), equilibrated with 50 mM Tris–HCl pH 8, 150 mM NaCl, 1 mM EDTA, 5 mM DTT, 20% glycerol (v/v). Elution was performed at 0.6 mL/min, and fractions of 0.9 mL were collected. The column was calibrated with LMW Gel Filtration Calibration kit (GE Healthcare). Pure PHP was eluted in dimeric and monomeric form, according to column calibration (S1 Fig). Monomeric PHP was concentrated, and stored at– 20°C until used.

### Determination of Protein Concentration

Protein concentration was determined according to Bradford [28]. Bovine serum albumin was used as standard.

### Enzyme Activity Assays

Steady-state activity assays were performed using a Perkin-Elmer (Waltham, MA, USA) λ19 spectrophotometer. Pyrophosphatase activity was assayed in the presence of 100 mM Tris-HCl (pH 8), 1 mM sodium pyrophosphate, 10 mM MgCl_2_, and 0.25 mM MnCl_2_. The reaction mixture did also contain 0.25 mM inosine, 50 mU/mL of Purine Nucleoside Phosphorylase (PNPase), and 500 mU/mL of Xanthine Oxidase (XOD) [29]. By this means, the released phosphate is used by PNPase for the phosphorolysis of inosine, generating hypoxanthine and ribose-1-phosphate. Hypoxanthine is then converted by XOD to uric acid, which can be conveniently monitored at 293 nm. For all the assays relying on the detection of uric acid we assumed 12,600 M^-1^cm^-1^ as the molar extinction coefficient for uric acid at 293 nm [30]. DNA Polymerase was assayed by detecting the pyrophosphate/phosphate released, in the presence or in the absence of 40 mU/mL of *E*. *coli* inorganic pyrophosphatase. For these assays, a dsDNA consisting of a 40mer template and a 15mer primer was used; this dsDNA features a 25mer polyA overhang [24]. Elongation of DNA (1 μM final concentration) was triggered by the addition of 100 μM dTTP, and the reaction kinetics was detected by monitoring uric acid at 293 nm. Steady-state 3’-5’ exonuclease activity was determined according to Hamdan et al. [31], using the 5’-*p*-nitrophenyl ester of thymidine monophosphate (pNP-TMP) as substrate. Reaction mixtures contained 3 mM pNP-TMP, 100 mM Tris-HCl (pH 8), 50 mM NaCl, 1 mM DTT, and 5 mM of a divalent cation (e.g. Mg^2+^), as indicated. Organic phosphatase activity was assayed in 100 mM Tris-HCl (pH 8), 50 mM NaCl, 1 mM DTT, using 1 mM *p*-nitrophenyl phosphate (PNP) as substrate, in the presence of 5 mM of a divalent cation, as indicated.

### Kinetic Analysis of Fluoride Inhibition of Pyrophosphatase Activity

To analyze the inhibition of fluoride towards *E*. *coli* inorganic PPase and PHP domain, a simple model was assumed: i) the binding of fluoride to enzyme is reversible, with k_1_ and k_2_ denoting the binding and dissociation rate constants, respectively; ii) the enzyme-F^-^ complex is inactive; iii) in each assay, the concentration of fluoride is constant (enzymes were used at concentrations at least 3 orders of magnitude lower than inhibitor concentration). Accordingly, the concentration of inactive enzyme ([E_I_]) is given by: [E_I_] = ([E_t_]•k_1_•[F]•(1 –e^-(k^_1_•^[F] + k^_2_^)^•^t^)/(k_1_•[F] + k_2_), where [E_t_] is the total enzyme concentration. Pyrophosphate concentration (1 mM) in the assays was assumed to be compatible with zero-order kinetics. Therefore, reaction velocity (d[P]/dt) is equal to k_cat_([E_t_]–[E_I_]). Upon integration, the following equation, used to fit the observed data, is obtained: [P] = V_max_•k_2_•t/(k_1_•[F] + k_2_) + (V_max_•k_1_•[F]/ (k_1_•[F] + k_2_)^2^)•(1 –e^- (k^_1_•^[F] + k^_2_^)^•^t^).

### FTIR Spectroscopy

Samples for FTIR spectroscopy were previously dried at 105°C for 48 h. Anhydrous KBr was added, and the mixture was homogenized with mortar and pestle. The samples in KBr were then subjected to 735 MPa, using a Perkin-Elmer hydraulic press. FTIR spectra were recorded with a Perkin-Elmer Spectrum One FTIR spectrometer.

### Microscopy

Pre-cultures of *E*. *coli* transformed with pBAD-α, pBAD-αH12A, or pBAD-αD201A were grown by picking single colonies from LB-ampicillin plates (0.1 mg/mL of antibiotic), and inoculating 2 mL of liquid LB-ampicillin medium. After overday growth at 37°C under shaking (180 rpm), the pre-cultures were diluted (1:500) in fresh LB-ampicillin medium, in the presence or in the absence of 1 mM arabinose. After 12 h of growth at 37°C, cells were collected by centrifugation, washed in PBS, and finally resuspended in the same buffer. To detect the fluorescence of nucleoids, Hoechst 33342 was added to the cells suspension. Micrographs were obtained with a Nikon Eclipse 600 microscope. To determine the size distribution of cells populations, the bright-field images were converted to 32-bit and processed with the ImageJ software [32]. In particular, each micrograph was subjected to bandpass filtering (2–40 pixel), and finally converted in binary format. The size of cells was determined, and, to avoid artifacts, individuals featuring an area smaller than 50 pixel^2^ were discarded. For each sample, 500 individuals were analyzed.

### Growth Kinetics and Mutations Frequency

Pre-cultures of the populations to be tested were obtained as described for the samples used for microscopy analysis. After overday growth at 37°C under shaking (180 rpm), pre-cultures were diluted (1:500) in Erlenmeyer flasks provided with a side-arm, in the presence or in the absence of 1 mM arabinose. Upon dilution, the Absorbance of the cells suspensions was determined at predetermined time intervals with a Biolog 21907 (Biolog, Hayward, CA, USA) turbidimeter. Mutations frequency was evaluated by plating aliquots of the bacterial populations on LB and on LB-rifampicin (500 μg/mL) plates. After incubation of the Petri dishes for 24 h, colonies were counted and the number of total and rifampicin-resistant individuals per unit volume was determined.

## Results and Discussion

### Pyrophosphatase Activity of the PHP Domain

**Fig 1:**
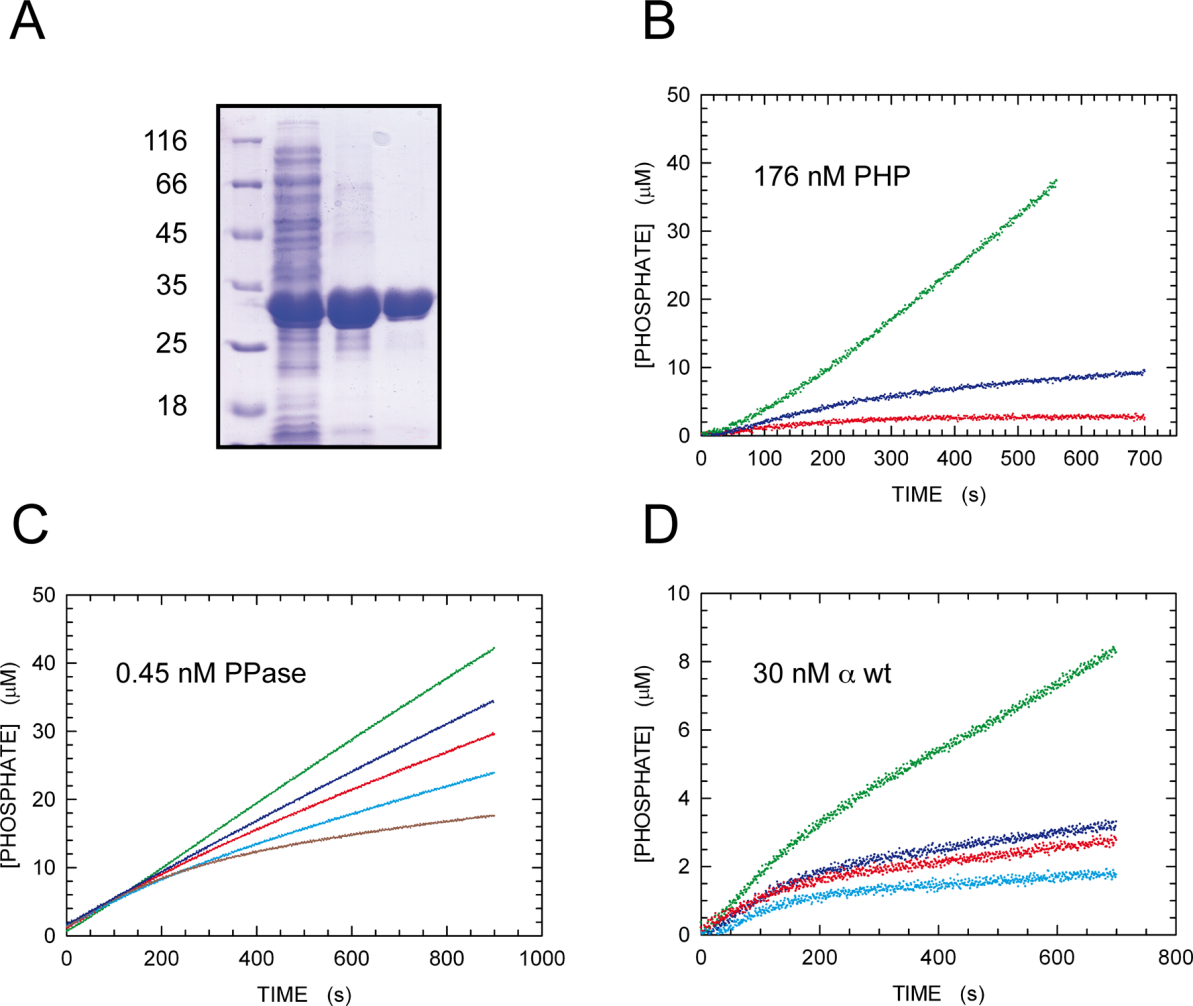
Purification and pyrophosphatase activity of PHP domain. **A)** SDS-PAGE of fractions collected during the purification of the *E*. *coli* PHP domain. M: molecular mass markers. Lane 1: soluble proteins isolated after refolding of the inclusion bodies containing PHP; lanes 2 and 3: purified PHP domain after ion exchange and gel filtration chromatography, respectively. **B)** Hydrolysis of pyrophospate by PHP domain. The release of orthophosphate as a function of time is reported for a complete assay mixture (green dots, 176 nM PHP and 1 mM pyrophosphate), and for assay solutions lacking pyrophosphate or enzyme (blue and red dots, respecitvely). **C)** Pyrophosphatase activity of 0.45 nM *E*. *coli* inorganic pyrophosphatase in the absence (green dots) or in the presence of 40, 100, 200, or 800 μM NaF (blue, red, cyano, and dark red dots, respectively). **D)** Pyrophosphatase activity of 30 nM full-length α subunit in the absence (green dots) or in the presence of 50, 200, or 800 μM NaF (blue, red, and cyano dots, respectively).

As a first test, we attempted to detect pyrophosphatase activity in the PHP domain. To this aim, we overexpressed from pBAD-α287 [23] the first N-terminal 287 residues of *E*. *coli* DNA Pol III α subunit, containing the whole PHP domain (amino acids 1–281). Using standard chromatographic techniques, we were able to isolate pure PHP in monomeric form (Fig 1A and S1 Fig), and we subjected the purified protein to pyrophosphatase activity assays.

**Fig 2:**
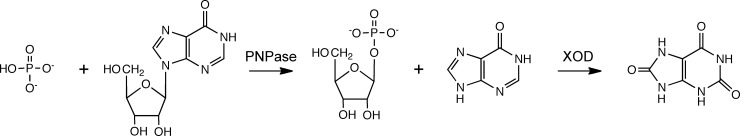
Reaction steps leading to the production of uric acid at the expense of phosphate and inosine.

These assays were performed according to our previously published procedure [29], relying on (Fig 2): i) the conversion of phosphate and inosine into ribose-1-phosphate and hypoxanthine, catalyzed by purine nucleoside phosphorylase (PNPase); ii) the oxidation of hypoxanthine into uric acid, catalyzed by xanthine oxidase (XOD); iii) the continuous detection of uric acid at 293 nm.

By this method, we observed consistent pyrophosphatase activity in the presence of PHP and 1 mM pyrophosphate, while in the absence of PHP or substrate the concentration of uric acid as a function of time did slighthly increase, presumably at the expense of contaminating phosphate (Fig 1B). Although we did not pursue a complete characterization of the pyrophosphatase activity of *E*. *coli* PHP domain, we estimated a k_obs_ (k_cat_ assuming [S] >>K_m_) equal to 0.4 s^-1^ for the hydrolysis of pyrophosphate catalyzed by PHP (Fig 1B). Notably, no activity was detected when the PHP domain was assayed for 3’-5’ exonuclease or organic phosphatase activity (S2 and S3 Figs).

According to the observations with PHP, we expected to detect pyrophosphatase activity in purified α subunit, and we asked if this activity was inhibited by fluoride ions. Therefore, we purified full-length wt α subunit, to be used for pyrophosphatase activity assays. As a comparison, we also performed similar assays with 0.45 nM *E*. *coli* inorganic pyrophosphatase (PPase). The activity of this enzyme obeyed zero-order kinetics in the absence of fluoride and, under these conditions, was equal to 47 nM/s (Fig 1C). When fluoride was present, inorganic PPase was inhibited, and the observed kinetics clearly deviated from zero-order (Fig 1C and S4 Fig). When 30 nM wt α subunit was assayed for pyrohosphatase activity in the absence of fluoride, the observed kinetics did obey zero-order for a limited amount of time, and was equal to 17.8 nM/s (Fig 1D). Therefore, when the concentrations of the assayed enzymes are considered, the activity of α subunit is about 180 times lower when compared to that of inorganic PPase. However, it should be mentioned that: i) we observed that the pyrophosphatase activity of α subunit is stimulated by Mn^2+^; ii) to avoid competition between fluoride and Mn^2+^, as reported for the pyrophosphatase from *Streptococcus gordonii* [33], we omitted Mn^2+^ in these assays. Notably, when fluoride was added to the assay mixture for α subunit, a strong inhibition of pyrophosphatase activity was observed, even at rather low concentrations of fluoride (Fig 1D and S5 Fig). Taking into account the reversible inhibition of pyrophosphatase activity by fluoride ions, we estimated the K_D_ for the fluoride-enzyme complex as equal to 146 ± 20 and 34 ± 22 μM for inorganic PPase and α subunit, respectively (S6 Fig). In addition, we estimated the residual pyrophosphatase activity of inorganic PPase and α subunit assuming that, 500 s after the reaction was started, a steady-state of the fluoride-enzyme complex was attained. The residual activity estimated by this method is in good agreement with the value for inactive enzyme concentration calculated at infinite time with the non-linear kinetics model (S6 Fig). Finally, the pyrophosphatase activity of α subunit was confirmed by FTIR spectroscopy. In the presence of α subunit, the band centered at 800 cm^-1^, known to arise from the P-O-P bond [34], was indeed observed to shift at 750 cm^-1^, as expected from a decrease in substrate concentration [34] (S7 and S8 Figs).

Overall, these observations indicate that *E*. *coli* DNA Pol III α subunit features pyrophosphatase activity, and the high sensitivity of this activity towards fluoride suggests the presence in this enzyme of a type-II PPase catalytic site [35].

### The Active Site of PHP Domain

**Fig 3:**
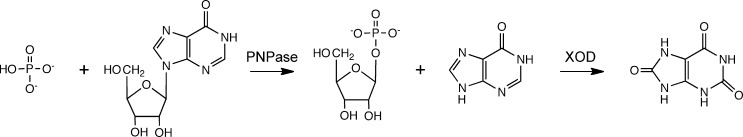
Structures of *E*. *coli* PHP and type-II inorganic PPases. **A)** Tertiary structure of *E*. *coli* DNA Polymerase III α subunit (PDB 2HNH). The PHP and the Polymerase domains are represented in green and blue, respectively. **B**,**C)** Detail of the entire PHP domain (**B**) and of the PHP β-sheet (**C**). The antiparallel β-strand is represented in **C** with green colour. **D**,**E)** Active sites of the type-II inorganic PPase from *Bacillus subtilis* (**D**, PDB 1WPM) and *Streptococcus gordonii* (**E**, PDB 1K20). The proposed active site of *E*. *coli* PHP is shown in panel **F**. The following amino acids are shown as sticks: H9, D13, D15, D75, H98, and D149 (**D**, *Bacillus subtilis*); H9, D13, D15, D77, H99, and D151 (**E**, *Streptococcus gordonii*); H12, D19, D43, D69, H83, and D201 (**F**, *Escherichia coli* PHP).

Type-II inorganic PPases feature active sites whose structure is significantly different from those of the well-known type-I enzymes [36]. Moreover, the activity of type-II enzymes depends on Mn^2+^ [36,37], in contrast to type-I PPases whose activity exclusively relies on Mg^2+^ [37]. To search the catalytic amino acids of α subunit involved in pyrophosphate hydrolysis, we analyzed the tertiary structure of the wt enzyme reported by Lamers et al. [22]. In particular, we focused our attention on the PHP region where an inorganic phosphate is bound, reasoning that this phosphate could represent the reaction product (Fig 3). In addition, we considered the active sites of 2 type-II inorganic PPases, namely from *Streptococcus gordonii* [38] and *Bacillus subtilis* [39]. In both these enzymes, a cluster of 4 Aspartates and a couple of Histidines are similarly oriented in the active site (Fig 3). By analogy, we hypothesized as members of the *E*. *coli* PHP domain active site H12, D19, D43, D69, H83, and D201 (Fig 3), and we decided to construct 3 site-specific mutants, i.e. H12A, D19A, and D201A. The vector pBAD-α1169 [23], containing wt *dnaE* (the gene coding for wt α subunit), was therefore used to obtain the desired variants. The addition of arabinose to the culture medium triggered high expression levels of each mutant (S9 Fig). However, the concentration of the D201A α subunit was rather low in the soluble fraction of protein extracts (S9 Fig). Therefore, we decided to purifiy the H12A and D19A variants, and to adopt a different strategy to obtain a purified form of αD201A. In particular, we co-expressed in *E*. *coli* the τ, α, ε, and θ subunits of DNA Pol III, taking advantage of our previously described co-expression system, based on the pBAD and pGOOD plasmids [24,40]. By this method, we readily obtained the τ_3_α_3_ε_3_θ_3_ complex [24], and we also overexpressed the τ_3_α(D201A)_3_ε_3_θ_3_ variant, the solubility of which was sufficient to permit its purification.

**Fig 4:**
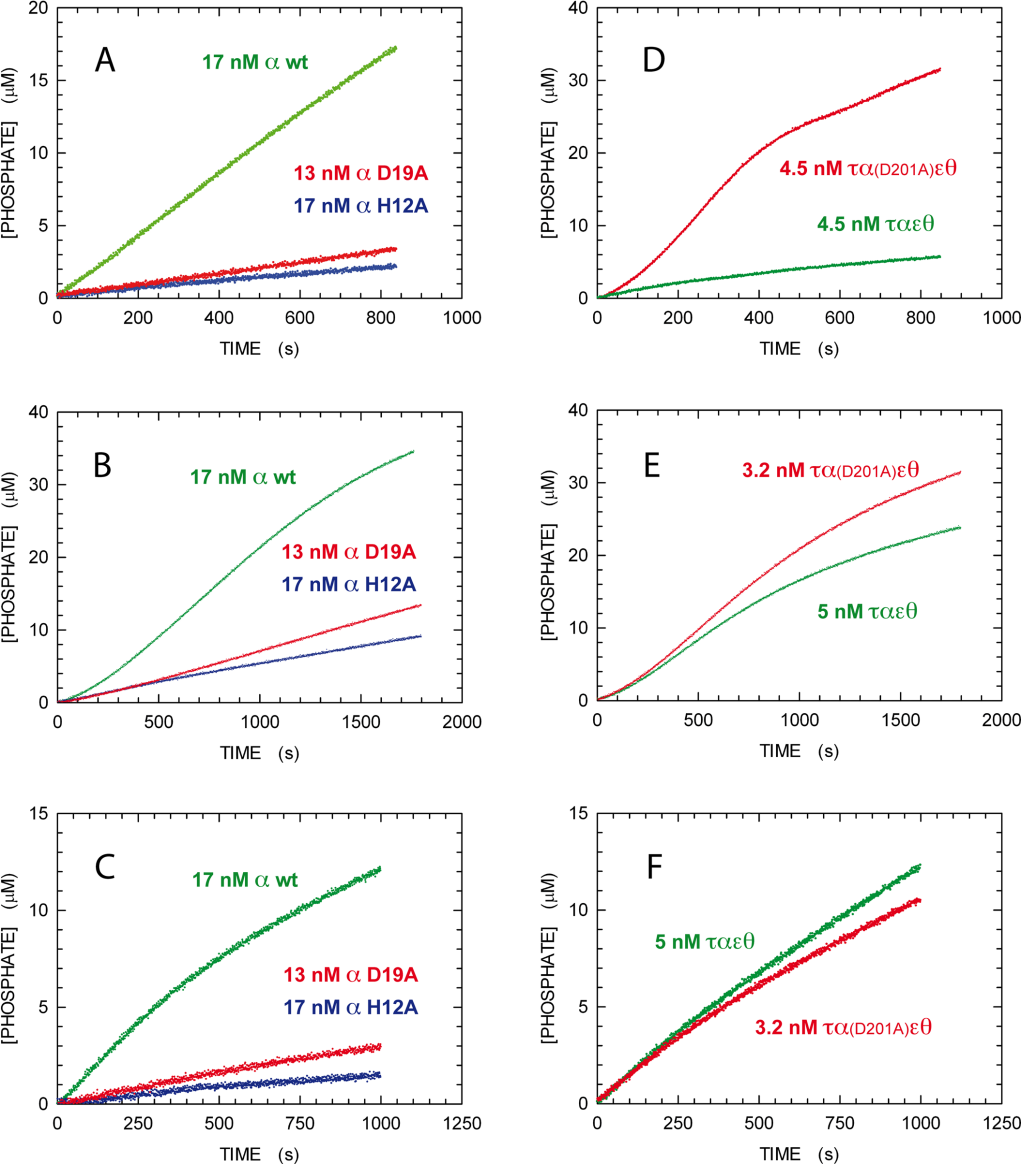
Pyrophosphatase and DNA Polymerase activity of wt and site-specific variants of α subunit. **A)** Pyrophosphatase activity of 17 nM wt (green dots), 17 nM H12A (blue dots), or 13 nM D19A (red dots) α subunit. **B**,**C)** DNA Polymerase activity as determined in the presence (**B**) or in the absence of *E*. *coli* inorganic pyrophosphatase (**C**). Other conditions as in **A**. **D)** Pyrophosphatase activity of 4.5 nM τ_3_α_3_ε_3_θ_3_ (green dots), or τ_3_α(D201A)_3_ε_3_θ_3_ complex (red dots). **E**,**F)** DNA Polymerase activity as determined in the presence (**E**) or in the absence of *E*. *coli* inorganic pyrophosphatase (**F**). The concentration of assayed enzymes were 5 and 3.2 nM for the wt and the τ_3_α(D201A)_3_ε_3_θ_3_ complex, respectively.

To test the importance of each mutated amino acid in the catalytic hydrolysis of pyrophosphate, we performed activity assays using 1 mM pyrophosphate as substrate, in the presence of 10 and 0.25 mM of MgCl_2_ and MnCl_2_, respectively. When wt α subunit was assayed at 17 nM, its activity was equal to 21 nM/s (Fig 4A). Under the same conditions, the activities observed in the presence of 17 and 13 nM of the H12A and D19A variants were determined as equal to 2.4 and 3.8 nM/s, respectively (Fig 4A). Accordingly, the corresponding k_obs_ are equal to 1.23, 0.14, and 0.29 s^-1^ for wt, H12A, and D19A α subunits, respectively. These observations strongly suggest the involvement of H12 and D19 in the hydrolysis of pyrophosphate catalyzed by the PHP domain.

Unexpectedly, when the pyrophosphatase activity of the τ_3_α_3_ε_3_θ_3_ complex was compared to that of the variant τ_3_α(D201A)_3_ε_3_θ_3_, the mutation at position 201 of α subunit was found to increase the catalytic efficiency. In particular, we observed reaction velocities equal to 12 and 63 nM/s in the presence of 4.5 nM wt or variant complex (Fig 4D), yielding k_obs_ equal to 2.7 and 14 s^-1^, respectively. Interestingly, the tertiary structure of wt α subunit indicates D201 as the closest amino acid to the phosphate ion bound to the PHP domain [22]. To explain the increase in activity due to the D201A substitution, we propose for D201 the role of gating the exit of the reaction product. The substitution of this aspartate with alanine would accordingly favour the release of phosphate, eventually preventing product inhibition of the PHP activity.

### Coupling PPase Activity and DNA Replication

Our enzyme-coupled activity assay could also be used to perform parallel tests of DNA Pol activity in the absence or in the presence of inorganic PPase (Fig 2). In the first case, the detection of uric acid is exclusively linked to the pyrophosphatase activity of PHP domain, while in the presence of an excess of inorganic PPase we observe the maximal rate of DNA extension. The comparison of these activities does therefore indicate the degree of coupling between DNA elongation and the intrinsic pyrophosphatase activity of the PHP domain. When 17 nM of the wt α subunit was tested in the presence of 1 μM DNA, we observed reaction velocities equal to 17.3 and 24.8 nM/s in the absence and presence of inorganic PPase, respectively (Fig 4C and 4B). Accordingly, the DNA Pol activity in the absence of PPase is 20–30% lower when compared with those determined using 1 mM pyrophosphate as substrate (21 nM/s, Fig 4A) or 1 μM DNA in the presence of excess PPase. These observations suggest that *E*. *coli* DNA Pol III α subunit features a significant, albeit not complete (70%), coupling between its DNA elongation and pyrophosphatase activities. To explain this coupling, we propose the presence in the α subunit of a pyrophosphate path connecting the palm (where pyrophosphate is generated) and the PHP domains. Accordingly, the pyrophosphate not channeled to the PHP domain would be released in solution, and would be consequently trapped and hydrolyzed by inorganic PPase. The coupling between DNA Pol and pyrophosphatase activities was confirmed by the assays performed with the variants H12A and D19A (at 17 and 13 nM). In fact, in the absence of inorganic PPase the reaction velocities were equal to 1.8 and 2.9 nM/s for H12A and D19A, respectively (Fig 4C). The corresponding activities observed in the presence of PPase were equal to 5.7 and 8 nM/s (Fig 4B). For both mutants, the coupling between pyrophosphatase and DNA Pol activity is about 35%, and this inefficient coupling triggers a decrease in the maximal rate of DNA elongation. When the τ_3_α_3_ε_3_θ_3_ complex was assayed at 5 nM, we observed reaction velocities equal to 14.7 and 19.9 nM/s in the absence and presence of inorganic PPase, respectively (Fig 4F and 4E). The corresponding velocities determined with 3.2 nM τ_3_α(D201A)_3_ε_3_θ_3_ were 13.2 and 24.5 nM/s (Fig 4F and 4E), indicating that the mutant complex features higher pyrophosphatase and DNA Pol activities.

**Fig 5:**
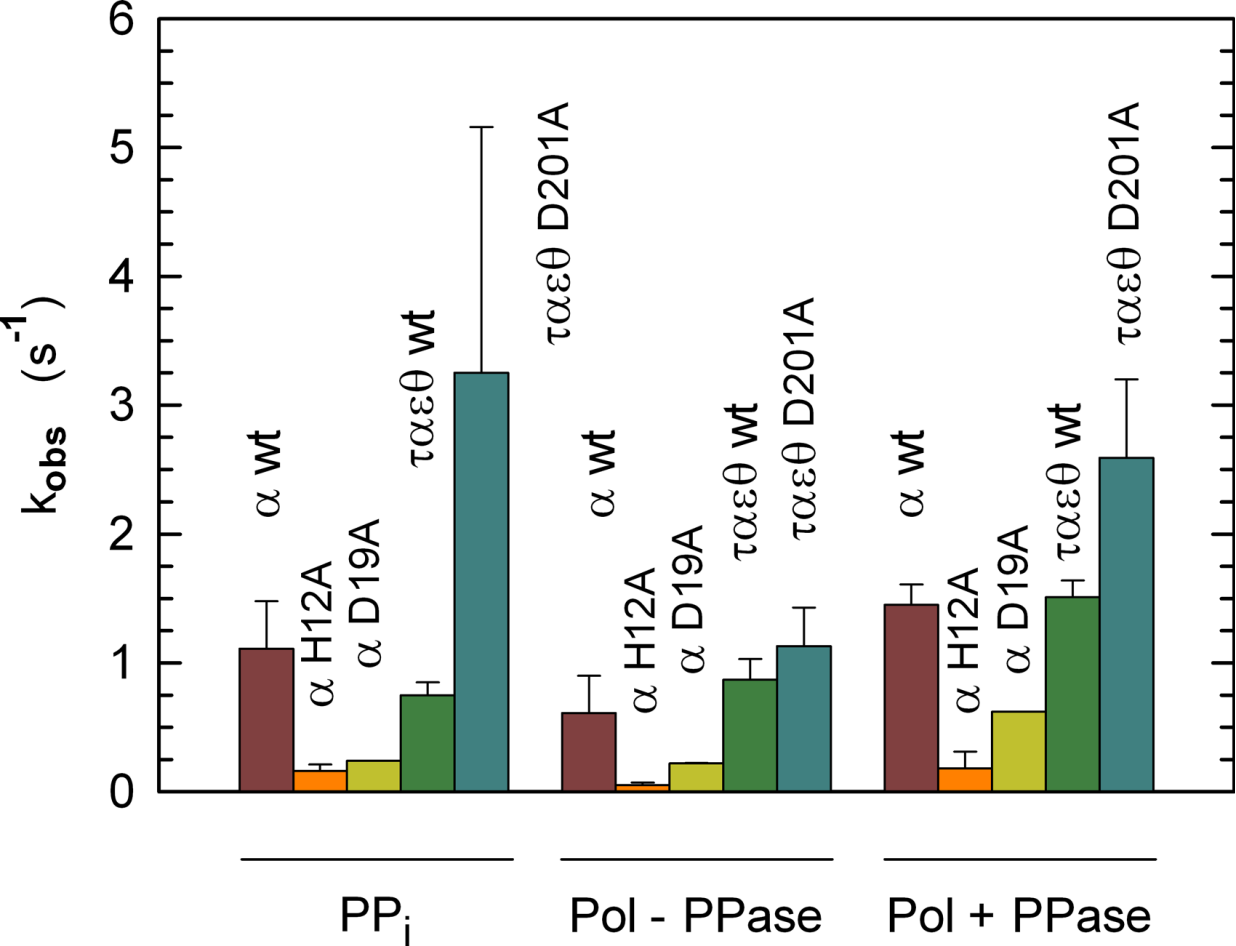
Observed rate constants of wt and site-specific variants of α subunit. Observed rate constants (v/[E_t_]) for the hydrolysis of pyrophosphate (PPi), and for DNA replication in the absence (Pol–PPase) or in the presence (Pol + PPase) of inorganic PPase. For the τ_3_α_3_ε_3_θ_3_ and τ_3_α(D201A)_3_ε_3_θ_3_ complex, rate constants are reported per monomer. The error bars represent, except for αD19A, the standard deviations of 3 independent kinetic assays, performed with different enzyme preparations. The purification yield of αD19A was not sufficient to perform triplicate assays. In this case, the error bars represent the standard deviations of the best linear fits to the different observed kinetics.

Overall, the catalytic properties of the PHP mutants indicate a strong coupling of the intrinsic pyrophosphatase and the DNA Pol activities of α subunit (Fig 5). We propose this coupling as a regulatory mechanism to control DNA elongation rate, and we thought of interest to test if altered couplings conferred a phenotype, if any, to *E*. *coli* individuals and populations.

### Altered PHP Activity and *E. coli* Phenotype

**Fig 6:**
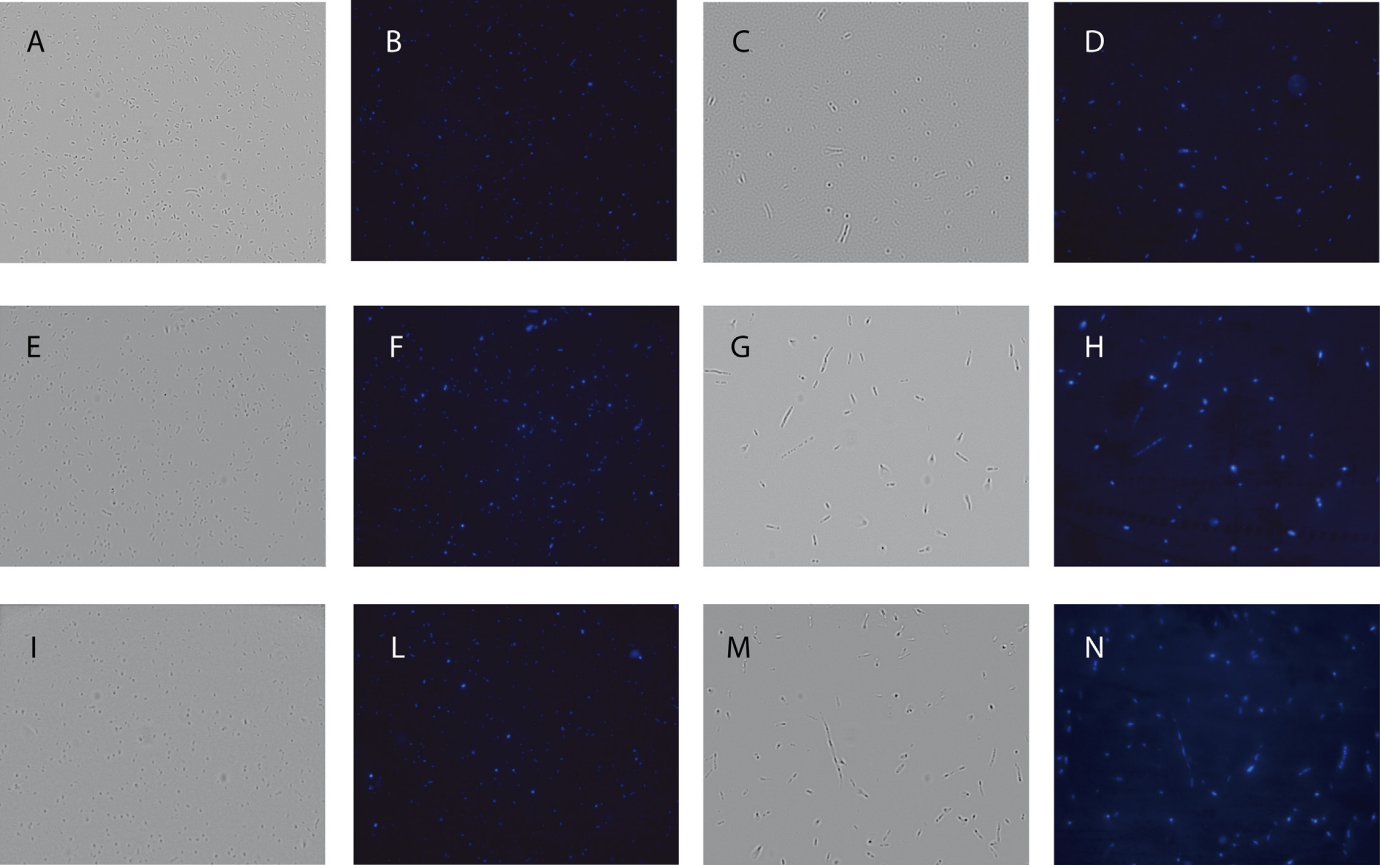
Phenotype of *E*. *coli* overexpressing or not the wt, D201A, or the H12A α subunit. Bright field (**A**,**C**,**E**,**G**,**I**,**M**) and fluorescence (**B**,**D**,**F**,**H**,**L**,**N**) micrographs of cells not-induced (**A**,**B**,**E**,**F**,**I**,**L**) or subjected (**C**,**D**,**G**,**H**,**M**,**N**) to overexpression of wt (**A**-**D**), D201A (**E**-**H**), or H12A (**I**-**N**) α subunit.

To test the effect of α subunit variants on *E*. *coli* phenotype, we overexpressed αD201A and αH12A, and we compared the morphology, the viability, and the mutation frequency of cells hosting these variants with those of individuals overexpressing the wt α subunit. In addition, not-induced cultures were used as controls. The induction of αD201A expression triggered aberrant phenotypes, characterized by filamented cells bearing multiple nucleoids (Fig 6 and S10 Fig). The overexpression of αH12A did also trigger aberrant morphologies, and the retention of a multiplicity of nucleoids in single cells (Fig 6 and S10 Fig).

**Fig 7:**
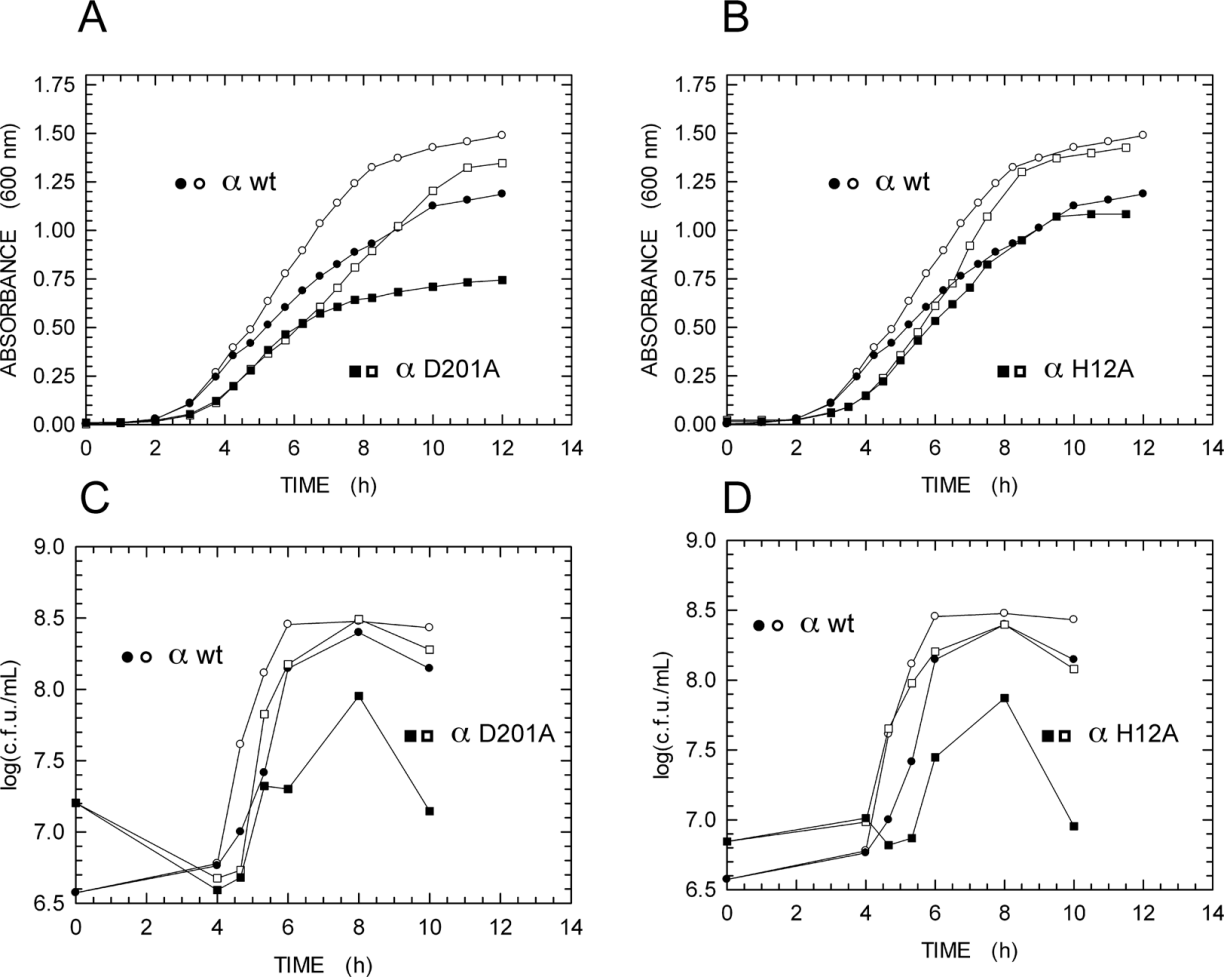
Growth kinetics of *E*. *coli* overexpressing or not the wt, D201A, or the H12A α subunit. **A**,**B)** Growth kinetics, determined spectroscopically, of *E*. *coli* populations overexpressing (filled symbols) or not (empty symbols) the wt (circles), D201A (squares, **A**), or the H12A (squares, **B**) α subunit. **C**,**D)** Growth kinetics, determined by colony counting, of *E*. *coli* populations overexpressing (filled symbols) or not (empty symbols) the wt (circles), D201A (squares, **C**), or the H12A (squares, **D**) α subunit.

We then decided to analyze the growth kinetics of *E*. *coli* populations hosting wt, D201A, or H12A α subunit. The overxepression of wt α subunit did slightly affect the viability of host cells (Fig 7A and 7B). On the contrary, severe growth defects were observed upon the induction of αD201A (Fig 7A and 7C) or αH12A (Fig 7B and 7D).

We therefore asked if the defective phenotypes conferred by the α subunit variants did also correspond to an increase in mutation frequency. In particular, we assayed with rifampicin-containing (0.5 mg/mL) Petri dishes, the frequency of appearance of Rif^R^ individuals in the different populations tested. We were able to determine quantitatively the mutation frequency of *E*. *coli* populations induced or not to the overexpression of wt α subunit, obtaining (6.7±5.9)•10^−8^ and (1.4±0.4)•10^−8^ values, respectively. On the contrary, when both not induced and induced cultures of *E*. *coli* hosting the D201A or the H12A variant were assayed, the mutation frequency was high enough to hamper a quantitative determination. We indeed observed, after culturing both mutants for a few generations, Rif^R^ clones at 10^−2^ frequency, the magnitude of which indicates a mutational load above the level compatible with viability [41,42].

In summary, the alteration of coupling between DNA elongation and pyrophosphatase activities of *E*. *coli* DNA Pol III α subunit dramatically affects the viability of cells, most likely because of an impairment of genome replication and cells growth. Remarkably, the viability of cells is strongly affected under conditions either decreasing (variant H12A) or increasing (variant D201A) the pyrophosphatase activity of the α subunit PHP domain.

## Concluding Remarks

Previous studies assigned to PHP domains from different bacterial species (*Deinococcus radiodurans*, *Bacillus subtilis*, *Thermus thermophilus*, *Streptococcus pneumoniae*, *Mycobacterium tuberculosis*) structural [21,43] or enzymatic functions [7,44–47], i.e. 3’-5’ exonuclease (proofreading) activity. Apparently in contrast with these observations, we report here that *E*. *coli* PHP domain features pyrophosphatase activity and is devoid of proofreading action (S2 Fig). However, our finding that *E*. *coli* PHP lacks 3’-5’ exonuclease activity is in agreement with a previous study [21]. Further, it was recently reported that replicative proofreading by PHP domains mainly occurs in bacterial species lacking the prototypical *E*. *coli* ε subunit [7], which exerts its exonucleolytic function towards the nucleotides misincorporated by the DNA Polymerase α subunit. Therefore, it is conceivable that different bacterial phylogenetic groups feature PHP domains bearing diverging functions. Accordingly, we propose that α-, β-, and γ-proteobacteria, sharing the expression of a *E*. *coli*-like ε subunit [7], do contain a PHP domain featuring pyrophosphatase activity and devoid of proofreading action.

We reported here that the overexpression of α subunits containing an inactivated (H12A) or an enhanced (D201A) PHP domain confers to *E*. *coli* aberrant morphology, poor viability, and mutator phenotype. These findings agree to previous observations with *Mycobacterium tuberculosis* [7], the growth rate and mutation frequency of which were severely altered by the expression of inactivated variants of PHP. Therefore, the relevance of PHP enzymatic activities for bacterial growth and genetic stability suggests this domain as a target for novel antibiotics.

Finally, it should be mentioned that we used a coupled assay to detect pyrophosphatase activity in *E*. *coli* PHP. In particular, the reaction product generated by PHP, orthophosphate, is continuously channeled with inosine into ribose-1-phosphate and hypoxanthine by the PNPase coupling enzyme. This prevents/alleviates inhibition of PHP by orthophosphate, which is known to inhibit *E*. *coli* inorganic pyrophosphatase either reversibly or irreversibly [48]. In particular, the irreversible inhibition of *E*. *coli* PPase by monoesters of phosphoric acid is well known [49]. Therefore, of remarkable interest could be the search for irreversible inhibitors of PHP, whose identification would disclose potential drugs directed towards proteobacteria.

## Supporting Information

S1 Fig **A** Gel filtration chromatography of *E*. *coli* Dna Polymerase III PHP domain. The best fractions eluted from a Q-Sepharose FF anion exchange column (1.6x25 cm) were pooled, concentrated to 1 mL and loaded onto a Superdex 200 column (1.6x70 cm). Elution was performed at 0.6 mL/min. The column was calibrated with Low-Molecular-Weight protein standards (GE Healthcare, USA). According to the calibartion of the column (y = 2.166–0.3543x, where y denotes K_AV_, and x is log of M_r_), fractions 44 and 54 contain proteins featuring molecular mass equal to 31.5 and 74 kDa, respectively. The expected molecular mass for *E*. *coli* PHP domain (amino acids 1–287 of α subunit) equals 31.7 kDa. **B** SDS-PAGE of fractions eluted from the Superdex 200 column. Fraction numbers are indicated at the top, and M denotes markers, whose molecular mass is reported at the left side.
(TIF)

S2 Fig Time-course of 3’-5’ exonuclease assays performed in the presence of 100 mM Tris-HCl pH 8, 50 mM NaCl, 1 mM DTT, 176 nM PHP domain, and 5 mM of the indicated divalent cation. 3 mM 5’-*p*-nitrophenyl ester of thymidine monophosphate (pNP-TMP) was used as substrate. The relase of product (*p*-nitrophenolate), if any, was determined at 420 nm.
(TIF)

S3 Fig Time-course of organic phosphatase assays performed in the presence of 100 mM Tris-HCl pH 8, 50 mM NaCl, 1 mM DTT, 176 nM PHP domain, and 5 mM of the indicated divalent cation. 1 mM *p*-nitrophenyl phosphate (pNP-P) was used as substrate. The relase of product (*p*-nitrophenolate), if any, was determined at 420 nm.
(TIF)

S4 Fig Kinetics of pyrophosphate hydrolysis catalyzed by inorganic 0.45 nM PPase in the presence of 40, 100, 200, 800, and 1200 μM NaF (panels A to E, respectively). Reactions were assayed in 100 mM Tris-HCl pH 8, 1 mM pyrophosphate, 5 mM MgCl_2_, 0.25 mM inosine, 50 mU/mL of Purine Nucleoside Phosphorylase (PNPase), and 500 mU/mL of Xanthine Oxidase (XOD). The time-course of Absorbance at 293 nm was determined, and the concentration of the released phosphate was calculated using a molar extinction coefficient for uric acid equal to 12,600 M^-1^cm^-1^. The continuous lines represent the best fits to the equation y = a + b•x + c•(1 –e^-d•x^), where a is the intercept with the y axis, b is V_max_•k_2_/(k_1_•[F] + k_2_), c is V_max_•k_1_•[F]/ (k_1_•[F] + k_2_)^2^, and d is k_1_•[F] + k_2_. The values of k_1_ and k_2_ (rate constants for the association of enzyme to fluoride, and for the dissociation of the PPase-F^-^ complex, respectively) accordingly obtained were used to determine the K_D_.
(TIF)

S5 Fig Kinetics of pyrophosphate hydrolysis catalyzed by 30 nM α subunit in the presence of 50, 200, 800, and 1200m μM NaF (panels A to D, respectively). Reactions were assayed in 100 mM Tris-HCl pH 8, 1 mM pyrophosphate, 10 mM MgCl_2_, 0.25 mM inosine, 50 mU/mL of Purine Nucleoside Phosphorylase (PNPase), and 500 mU/mL of Xanthine Oxidase (XOD). The time-course of Absorbance at 293 nm was determined, and the concentration of the released phosphate was calculated using a molar extinction coefficient for uric acid equal to 12,600 M^-1^cm^-1^. The continuous lines represent the best fits to the equation y = a + bx + c•(1 –e^-d•x^), where a is the intercept with the y axis, b is V_max_•k_2_/(k_1_•[F] + k_2_), c is V_max_•k_1_•[F]/ (k_1_•[F] + k_2_)^2^, and d is k_1_•[F] + k_2_. The values of k_1_ and k_2_ (rate constants for the association of enzyme to fluoride, and for the dissociation of the α subunit-F^-^ complex, respectively) accordingly obtained were used to determine the K_D_.
(TIF)

S6 Fig **A** K_**D**_ of the PPase-F^-^ and α subunit-F^-^ complexes as determined with the kinetics of pyrophosphate hydrolysis assayed in the presence of different concentrations of NaF (S4 and S5 Figs). **B**,**C** Residual pyrophosphatase activity (filled circles) and inactivation (empty circles) of inorganic PPase (**B**) or α subunit (**C**). Assuming that 500 s after the reaction was started the concentration of enzyme-F^-^ complex reached a steady-state, the residual activity was considered as the ratio of reaction velocity in the 500–700 s time interval over initial velocity. The concentration of inactive enzyme (E-F^-^ complex) at infinite time was calculated according to: [E_I_] = ([E_t_]•k_1_•[F])/(k_1_•[F] + k_2_), where [E_t_] is the total enzyme concentration.
(TIF)

S7 Fig FTIR spectra of Mg-orthophosphate and of Mg-pyrophosphate. Solid MgHPO_4_ was extensively dried at 105°C, mixed with KBr, and the mixture was subjected to 735 MPa. FTIR spectra were immediately acquired using a Perkin-Elmer Spectrum One spectrometer. To prepare Mg-pyrophosphate, a reaction mixture containing 100 mM Tris-HCl pH 8, 1 mM sodium pyrophosphate, 10 mM MgCl_2_, was incubated in the absence of any added enzyme. After 3 h, the suspension was centrifuged (10,000xg, 20 min), and the supernatant was discarded. The pellet, containing Mg-pyrophosphate, was dried and processed as previosuly mentioned for Mg-orthophosphate. **A** FTIR spectra of Mg-pyrophosphate (blue line) and of Mg-orthophosphate (green line) over a 4000–450 cm^-1^ wavenumber interval. **B** Superimposed FTIR spectra of mixtures containing 15 and 85, 25 and 75, 35 and 65, 50 and 50% of Mg-orthophosphate and Mg-pyrophosphate (green, blue, red, and pink lines, respectively). **C**,**D** Details of the spectra reported in **A**,**B** over a 1050–450 cm^-1^ wavenumber interval. In **C** the wavenumbers of the most relevant bands are indicated. It should be noted that the lowest-energy bands of Mg-pyrophosphate and Mg-orthophosphate are centered at 471 and 507 cm^-1^, respectively. The bands at 747 and 800 cm^-1^ are known to be generated by the P-O-P bond [34]. In **D** the FTIR spectrum of Mg-pyrophosphate (black line) is also reported for comparison. By this means, the progressive shift towards 502 cm^-1^ of the Mg-pyrophosphate band centered at 471 cm^-1^ is clearly visible as a function of Mg-orthophosphate concentration (cf. with panel **C**).
(TIF)

S8 Fig FTIR spectra of residual substrate and product of reactions catalyzed by inorganic PPase (**A**) and PHP domain (**B**). **A** A reaction mixture containing 100 mM Tris-HCl pH 8, 10 mM MgCl_2_, 0.25 mM MnCl_2_, 1 mM Na_4_P_2_O_7_, and 2 nM inorganic PPase, was incubated for 15 h at room temperature. The solution was then centrifuged (10,000xg, 20 min), the supernatant was discarded, and the pellet was dried and processed with KBr to perform FTIR spectroscopy. **B** Reaction mixtures containing 100 mM Tris-HCl pH 8, 10 mM MgCl_2_, 0.25 mM MnCl_2_, 1 mM Na_4_P_2_O_7_, and 100 or 300 nM α subunit (green and blue lines, respectively), were incubated for 15 h at room temperature. The solutions were then centrifuged (10,000xg, 20 min), the supernatants were discarded, and the pellets were dried and processed with KBr to perform FTIR spectroscopy. **C**,**D** Details of the spectra reported in **A**,**B** over a 1050–450 cm^-1^ wavenumber interval. In **C** (PPase-catalyzed reaction) the absence and the shift, respectively, of the Mg-pyrophosphate bands at 800 and 471 cm^-1^ can be clearly noted (cf. S7 Fig). Similarly, in **D** a strong decrease and a significant shift of the bands at 800 and 471 cm^-1^ are recognized, as a function of α subunit concentration.
(TIF)

S9 Fig **A** SDS-PAGE of total proteins extracted from *E*. *coli* TOP10 overexpressing wt α subunit, or the D201A, H12A, and D19A variants (lanes 2, 4, 6, and 8, respectively). The corresponding protein extracts isolated from *E*. *coli* cultures not subjected to overexpression are reported in lanes 1, 3, 5, and 7. **B** SDS-PAGE of soluble proteins extracted from *E*. *coli* TOP10 overexpressing wild-type α subunit, or the D201A variant (lanes 2 and 4, respectively). The corresponding protein extracts isolated from *E*. *coli* cultures not subjected to overexpression are reported in lanes 1 and 3.
(TIF)

S10 Fig Size distribution of *E*. *coli* populations not induced (**A**,**C**,**E**) or induced (**B**,**D**,**F**) to overexpress wt α subunit (**A**,**B**), the D201A (**C**,**D**) or the H12A (**E**,**F**) variant. Bright-field micrographs of the different samples were acquired with a Nikon Eclipse 600 microscope. The images accordingly obtained were processed with the ImageJ software and, upon their conversion in binary format, cells area was determined. For every population 500 individuals were considered, and the sum of cells featuring area < 200 pixel^2^ is indicated in each panel.
(TIF)
