# Correction: *Escherichia coli* DnaE Polymerase Couples Pyrophosphatase Activity to DNA Replication

**DOI:** 10.1371/journal.pone.0157207

**Published:** 2016-06-06

**Authors:** The PLOS ONE Staff

Fig 3 is incorrect. The authors have provided a corrected version here. The publisher apologizes for the error.

**Fig 3:**
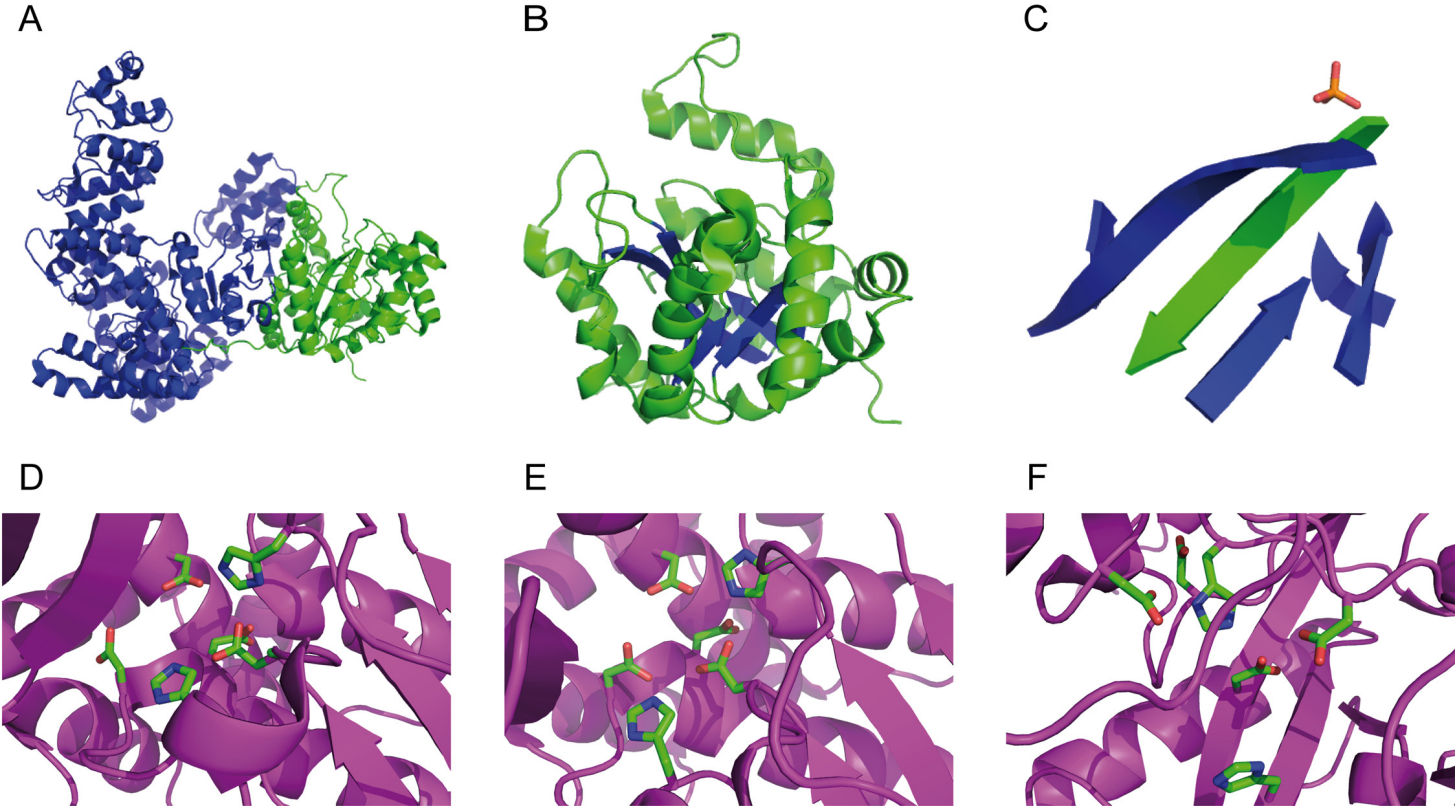
Structures of E. coli PHP and type-II inorganic PPases. A) Tertiary structure of E. coli DNA Polymerase III α subunit (PDB 2HNH). The PHP and the Polymerase domains are represented in green and blue, respectively. B,C) Detail of the entire PHP domain (B) and of the PHP β-sheet (C). The antiparallel β-strand is represented in C with green colour. D,E) Active sites of the type-II inorganic PPase from Bacillus subtilis (D, PDB 1WPM) and Streptococcus gordonii (E, PDB 1K20). The proposed active site of E. coli PHP is shown in panel F. The following amino acids are shown as sticks: H9, D13, D15, D75, H98, and D149 (D, Bacillus subtilis); H9, D13, D15, D77, H99, and D151 (E, Streptococcus gordonii); H12, D19, D43, D69, H83, and D201 (F, Escherichia coli PHP).
