# Supplementary figures and images for: Escherichia coli DnaE Polymerase Couples Pyrophosphatase Activity to DNA Replication
Source: PLoS One. 2016 Apr 6;11(4):e0152915. doi: 10.1371/journal.pone.0152915 (PMC4822814; doi:10.1371/journal.pone.0152915)

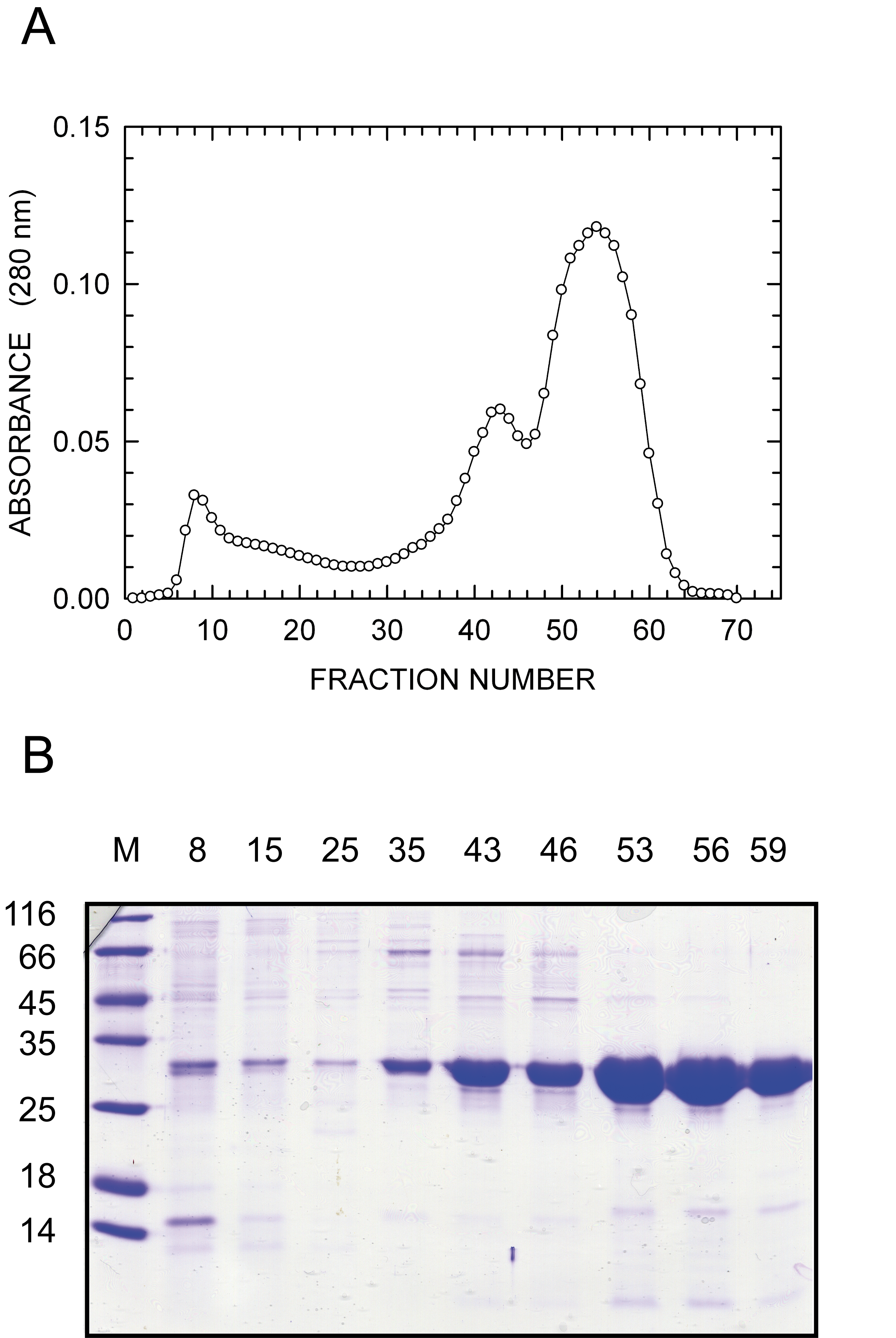

Supplement: S1 Fig — A Gel filtration chromatography of E. coli Dna Polymerase III PHP domain. The best fractions eluted from a Q-Sepharose FF anion exchange column (1.6x25 cm) were pooled, concentrated to 1 mL and loaded onto a Superdex 200 column (1.6x70 cm). Elution was performed at 0.6 mL/min. The column was calibrated with Low-Molecular-Weight protein standards (GE Healthcare, USA). According to the calibartion of the column (y = 2.166–0.3543x, where y denotes KAV, and x is log of Mr), fractions 44 and 54 contain proteins featuring molecular mass equal to 31.5 and 74 kDa, respectively. The expected molecular mass for E. coli PHP domain (amino acids 1–287 of α subunit) equals 31.7 kDa. B SDS-PAGE of fractions eluted from the Superdex 200 column. Fraction numbers are indicated at the top, and M denotes markers, whose molecular mass is reported at the left side. (TIF) [file pone.0152915.s001.tif]

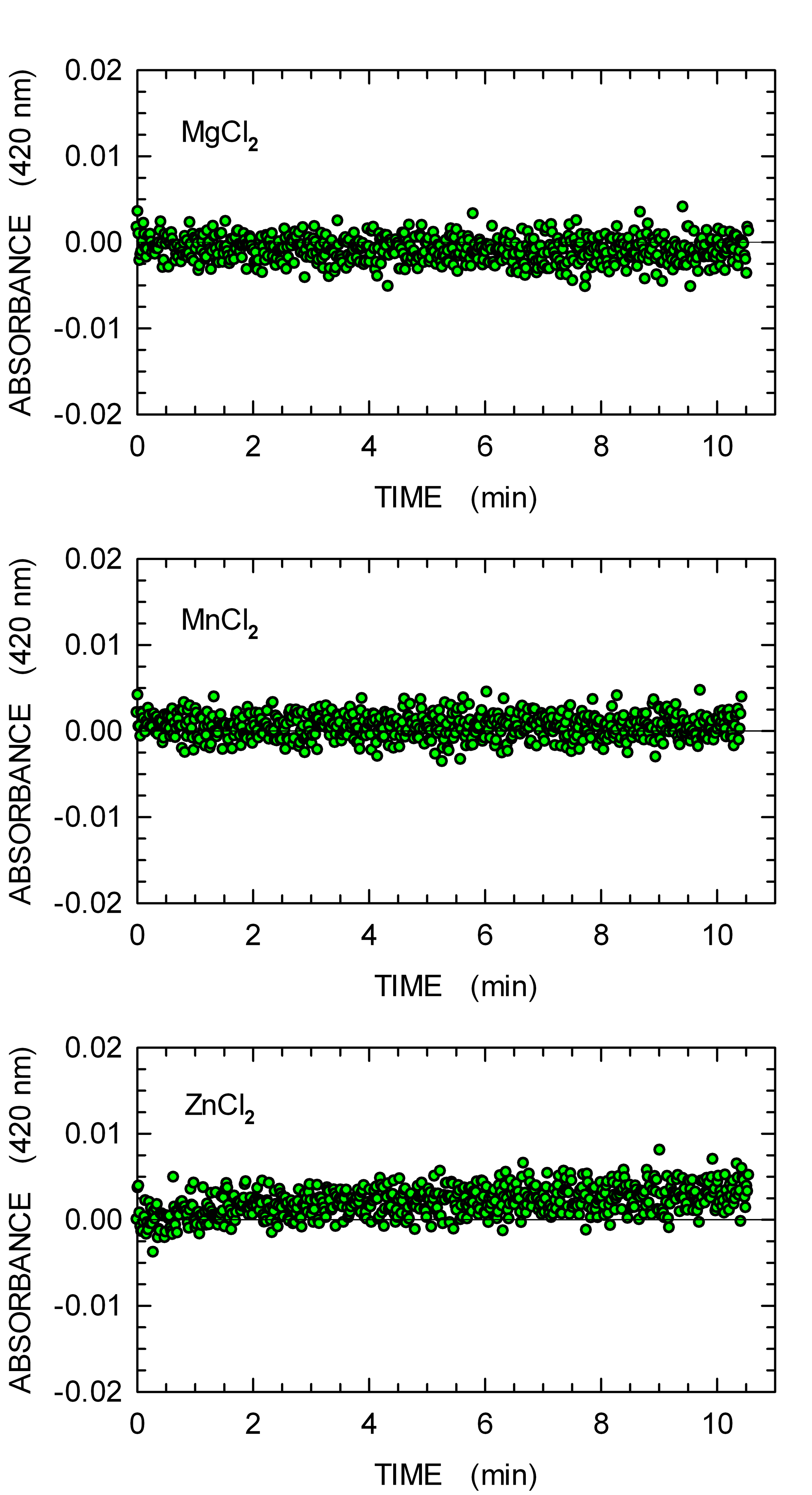

Supplement: S2 Fig — 3 mM 5’-p-nitrophenyl ester of thymidine monophosphate (pNP-TMP) was used as substrate. The relase of product (p-nitrophenolate), if any, was determined at 420 nm. (TIF) [file pone.0152915.s002.tif]

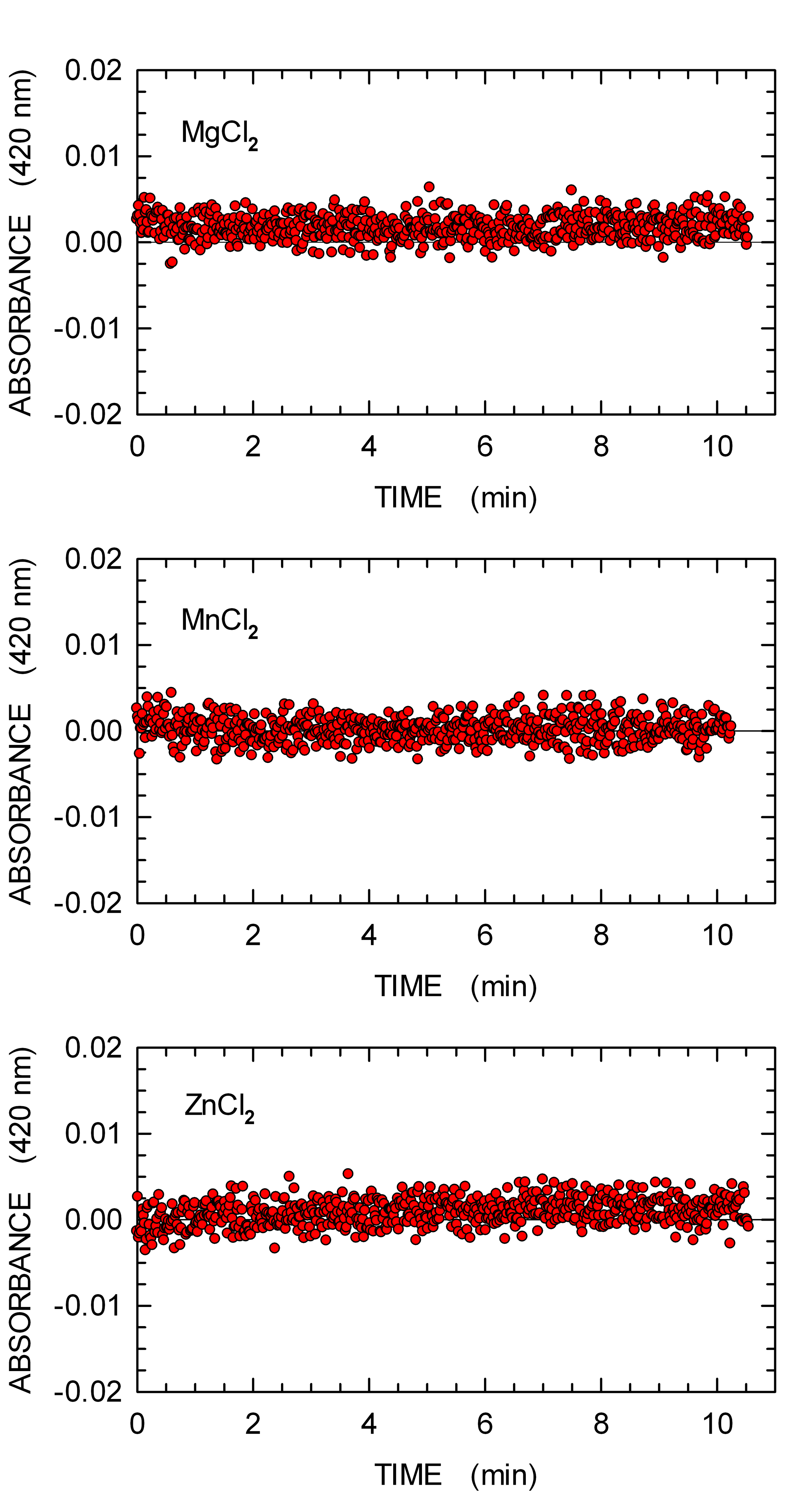

Supplement: S3 Fig — 1 mM p-nitrophenyl phosphate (pNP-P) was used as substrate. The relase of product (p-nitrophenolate), if any, was determined at 420 nm. (TIF) [file pone.0152915.s003.tif]

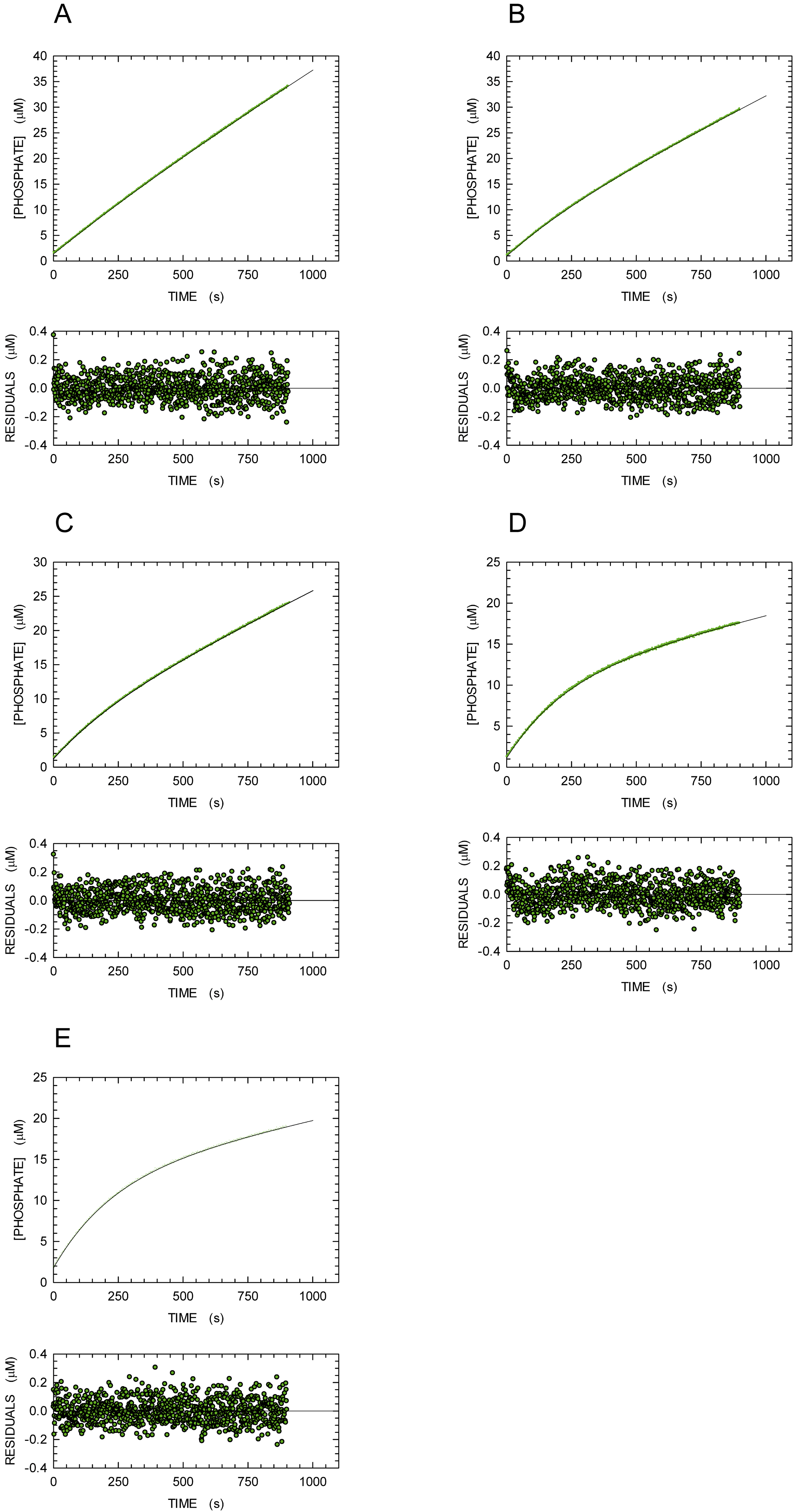

Supplement: S4 Fig — Reactions were assayed in 100 mM Tris-HCl pH 8, 1 mM pyrophosphate, 5 mM MgCl2, 0.25 mM inosine, 50 mU/mL of Purine Nucleoside Phosphorylase (PNPase), and 500 mU/mL of Xanthine Oxidase (XOD). The time-course of Absorbance at 293 nm was determined, and the concentration of the released phosphate was calculated using a molar extinction coefficient for uric acid equal to 12,600 M-1cm-1. The continuous lines represent the best fits to the equation y = a + b•x + c•(1 –e-d•x), where a is the intercept with the y axis, b is Vmax•k2/(k1•[F] + k2), c is Vmax•k1•[F]/ (k1•[F] + k2)2, and d is k1•[F] + k2. The values of k1 and k2 (rate constants for the association of enzyme to fluoride, and for the dissociation of the PPase-F- complex, respectively) accordingly obtained were used to determine the KD. (TIF) [file pone.0152915.s004.tif]

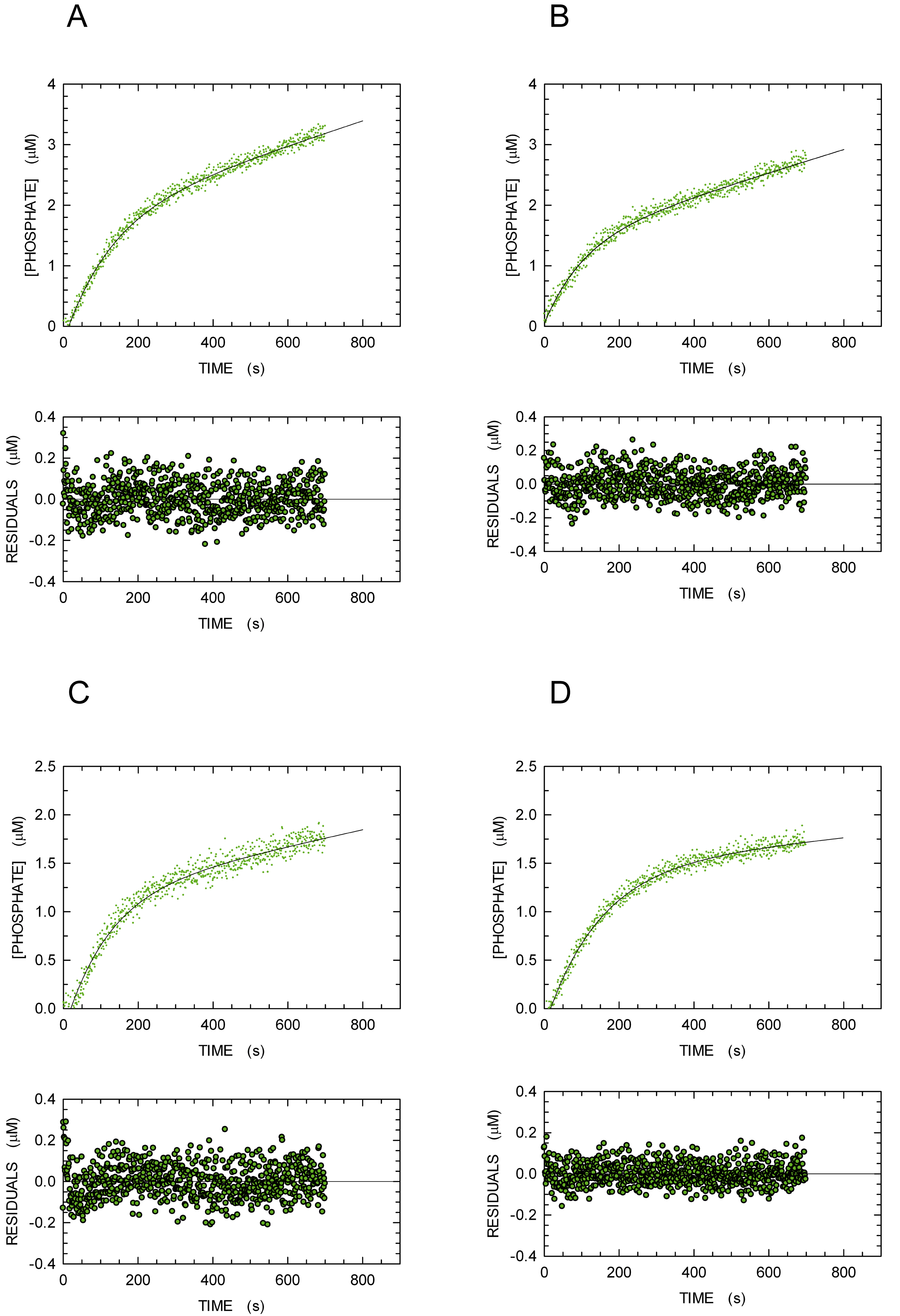

Supplement: S5 Fig — Reactions were assayed in 100 mM Tris-HCl pH 8, 1 mM pyrophosphate, 10 mM MgCl2, 0.25 mM inosine, 50 mU/mL of Purine Nucleoside Phosphorylase (PNPase), and 500 mU/mL of Xanthine Oxidase (XOD). The time-course of Absorbance at 293 nm was determined, and the concentration of the released phosphate was calculated using a molar extinction coefficient for uric acid equal to 12,600 M-1cm-1. The continuous lines represent the best fits to the equation y = a + bx + c•(1 –e-d•x), where a is the intercept with the y axis, b is Vmax•k2/(k1•[F] + k2), c is Vmax•k1•[F]/ (k1•[F] + k2)2, and d is k1•[F] + k2. The values of k1 and k2 (rate constants for the association of enzyme to fluoride, and for the dissociation of the α subunit-F- complex, respectively) accordingly obtained were used to determine the KD. (TIF) [file pone.0152915.s005.tif]

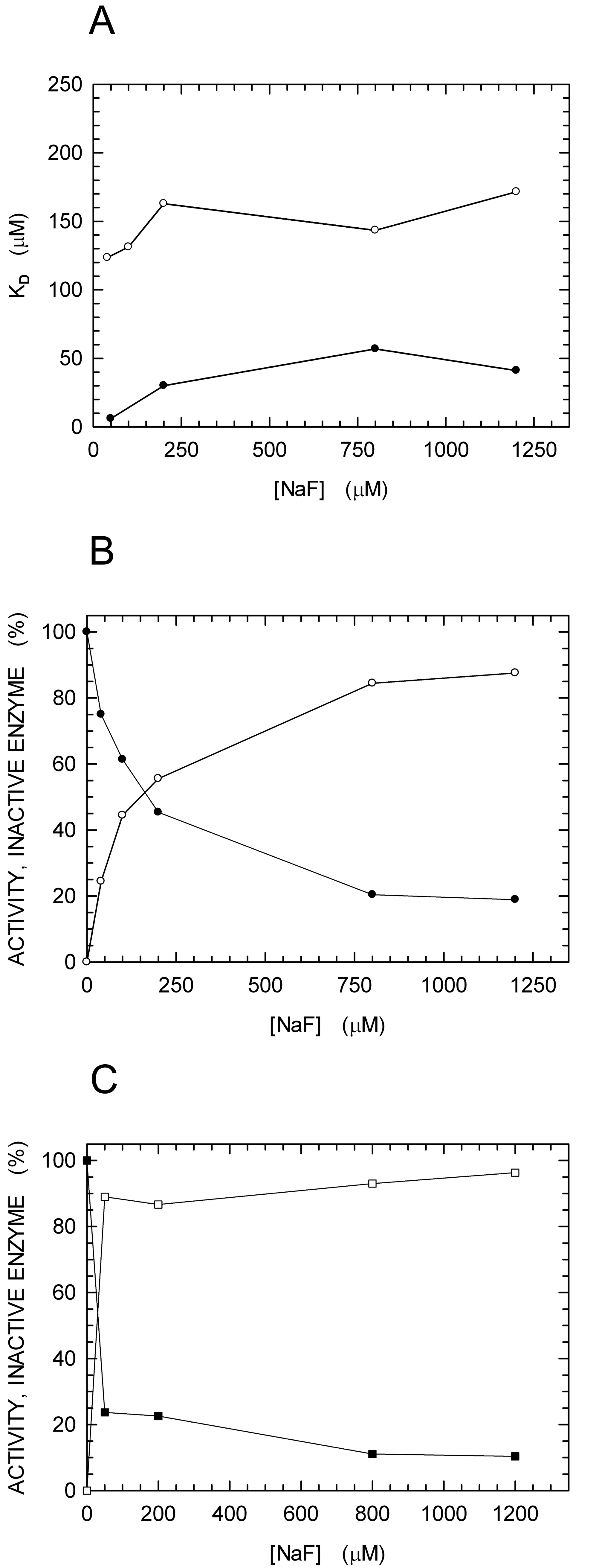

Supplement: S6 Fig — A KD of the PPase-F- and α subunit-F- complexes as determined with the kinetics of pyrophosphate hydrolysis assayed in the presence of different concentrations of NaF (S4 and S5 Figs). B,C Residual pyrophosphatase activity (filled circles) and inactivation (empty circles) of inorganic PPase (B) or α subunit (C). Assuming that 500 s after the reaction was started the concentration of enzyme-F- complex reached a steady-state, the residual activity was considered as the ratio of reaction velocity in the 500–700 s time interval over initial velocity. The concentration of inactive enzyme (E-F- complex) at infinite time was calculated according to: [EI] = ([Et]•k1•[F])/(k1•[F] + k2), where [Et] is the total enzyme concentration. (TIF) [file pone.0152915.s006.tif]

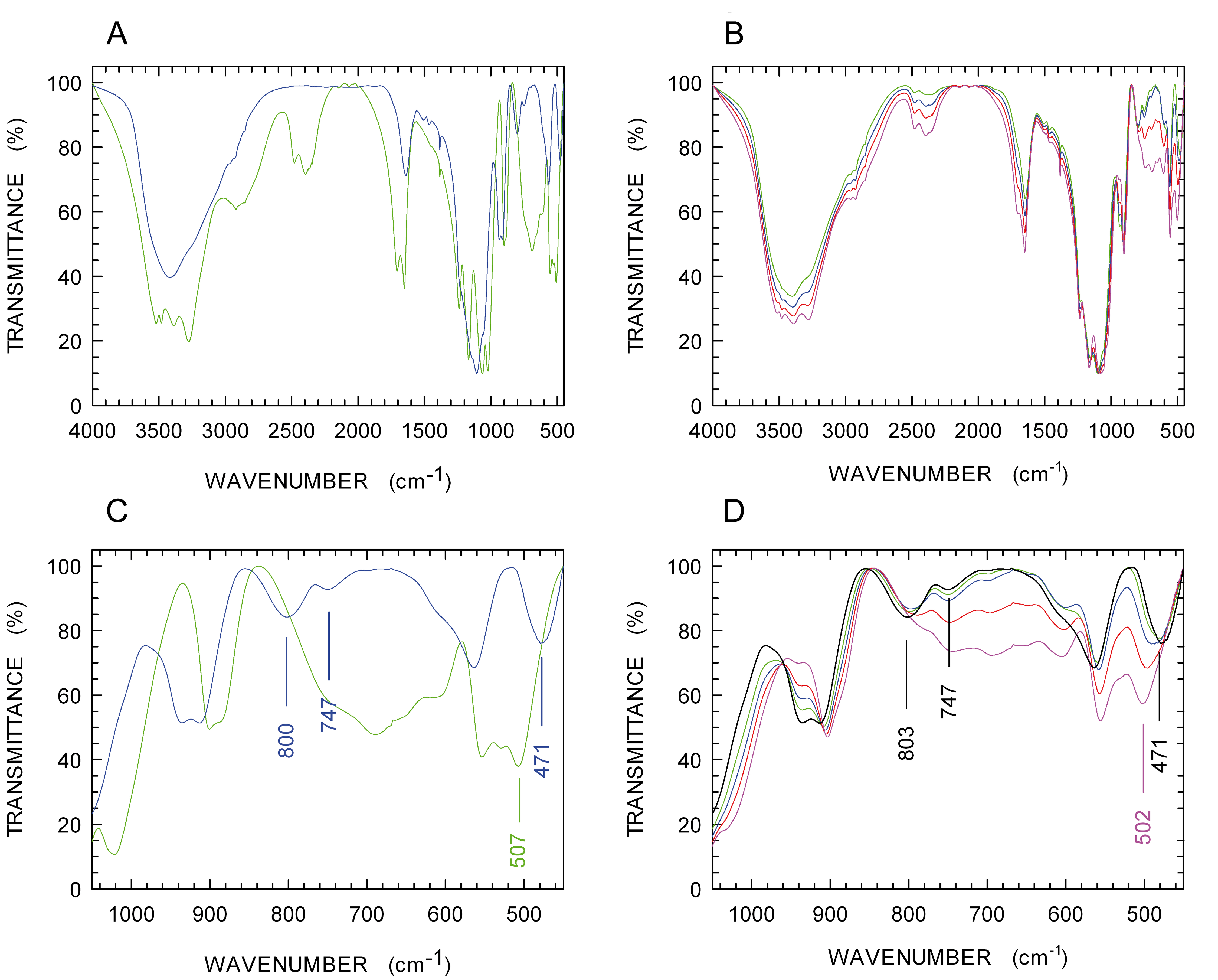

Supplement: S7 Fig — Solid MgHPO4 was extensively dried at 105°C, mixed with KBr, and the mixture was subjected to 735 MPa. FTIR spectra were immediately acquired using a Perkin-Elmer Spectrum One spectrometer. To prepare Mg-pyrophosphate, a reaction mixture containing 100 mM Tris-HCl pH 8, 1 mM sodium pyrophosphate, 10 mM MgCl2, was incubated in the absence of any added enzyme. After 3 h, the suspension was centrifuged (10,000xg, 20 min), and the supernatant was discarded. The pellet, containing Mg-pyrophosphate, was dried and processed as previosuly mentioned for Mg-orthophosphate. A FTIR spectra of Mg-pyrophosphate (blue line) and of Mg-orthophosphate (green line) over a 4000–450 cm-1 wavenumber interval. B Superimposed FTIR spectra of mixtures containing 15 and 85, 25 and 75, 35 and 65, 50 and 50% of Mg-orthophosphate and Mg-pyrophosphate (green, blue, red, and pink lines, respectively). C,D Details of the spectra reported in A,B over a 1050–450 cm-1 wavenumber interval. In C the wavenumbers of the most relevant bands are indicated. It should be noted that the lowest-energy bands of Mg-pyrophosphate and Mg-orthophosphate are centered at 471 and 507 cm-1, respectively. The bands at 747 and 800 cm-1 are known to be generated by the P-O-P bond [34]. In D the FTIR spectrum of Mg-pyrophosphate (black line) is also reported for comparison. By this means, the progressive shift towards 502 cm-1 of the Mg-pyrophosphate band centered at 471 cm-1 is clearly visible as a function of Mg-orthophosphate concentration (cf. with panel C). (TIF) [file pone.0152915.s007.tif]

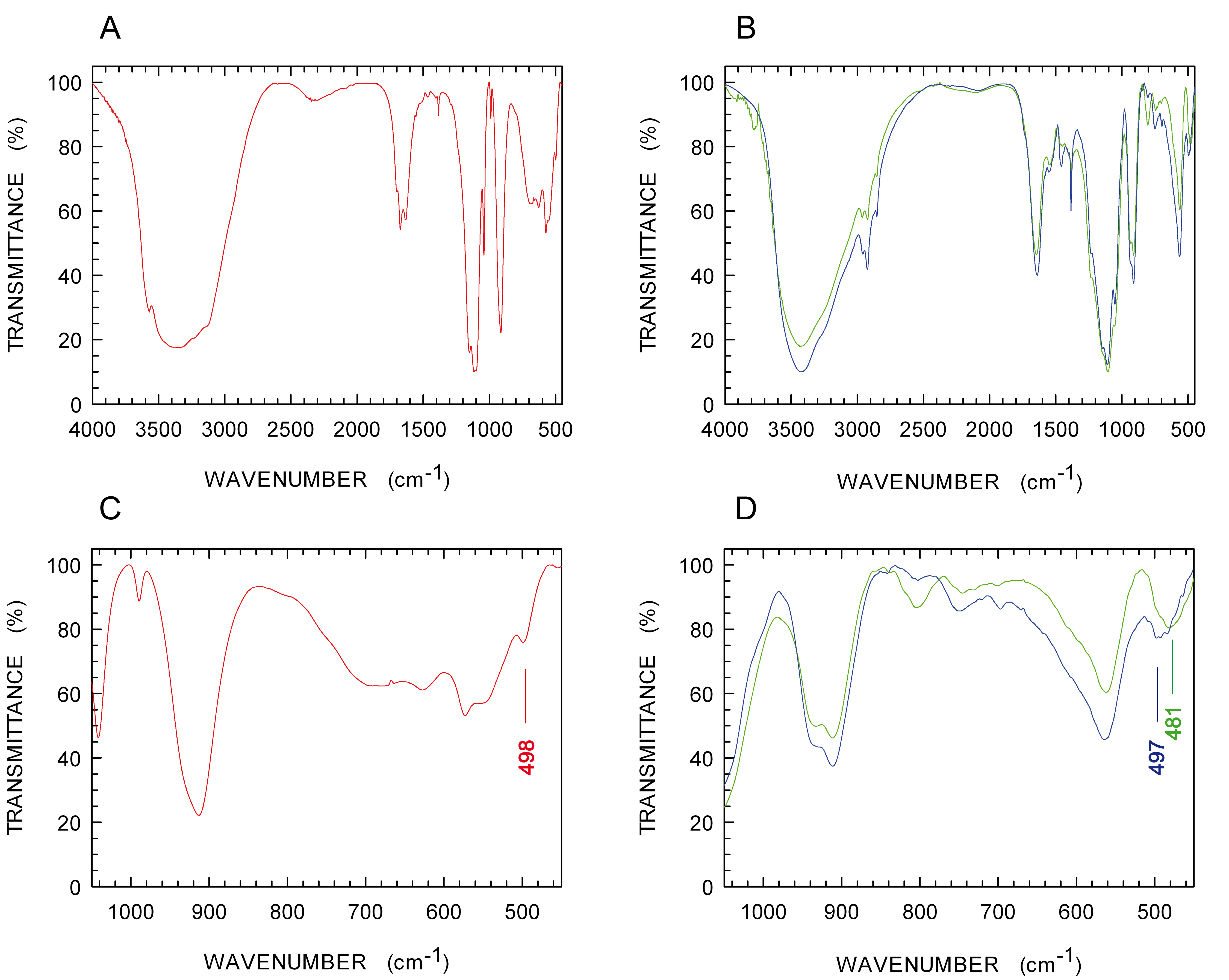

Supplement: S8 Fig — FTIR spectra of residual substrate and product of reactions catalyzed by inorganic PPase (A) and PHP domain (B). A A reaction mixture containing 100 mM Tris-HCl pH 8, 10 mM MgCl2, 0.25 mM MnCl2, 1 mM Na4P2O7, and 2 nM inorganic PPase, was incubated for 15 h at room temperature. The solution was then centrifuged (10,000xg, 20 min), the supernatant was discarded, and the pellet was dried and processed with KBr to perform FTIR spectroscopy. B Reaction mixtures containing 100 mM Tris-HCl pH 8, 10 mM MgCl2, 0.25 mM MnCl2, 1 mM Na4P2O7, and 100 or 300 nM α subunit (green and blue lines, respectively), were incubated for 15 h at room temperature. The solutions were then centrifuged (10,000xg, 20 min), the supernatants were discarded, and the pellets were dried and processed with KBr to perform FTIR spectroscopy. C,D Details of the spectra reported in A,B over a 1050–450 cm-1 wavenumber interval. In C (PPase-catalyzed reaction) the absence and the shift, respectively, of the Mg-pyrophosphate bands at 800 and 471 cm-1 can be clearly noted (cf. S7 Fig). Similarly, in D a strong decrease and a significant shift of the bands at 800 and 471 cm-1 are recognized, as a function of α subunit concentration. (TIF) [file pone.0152915.s008.tif]

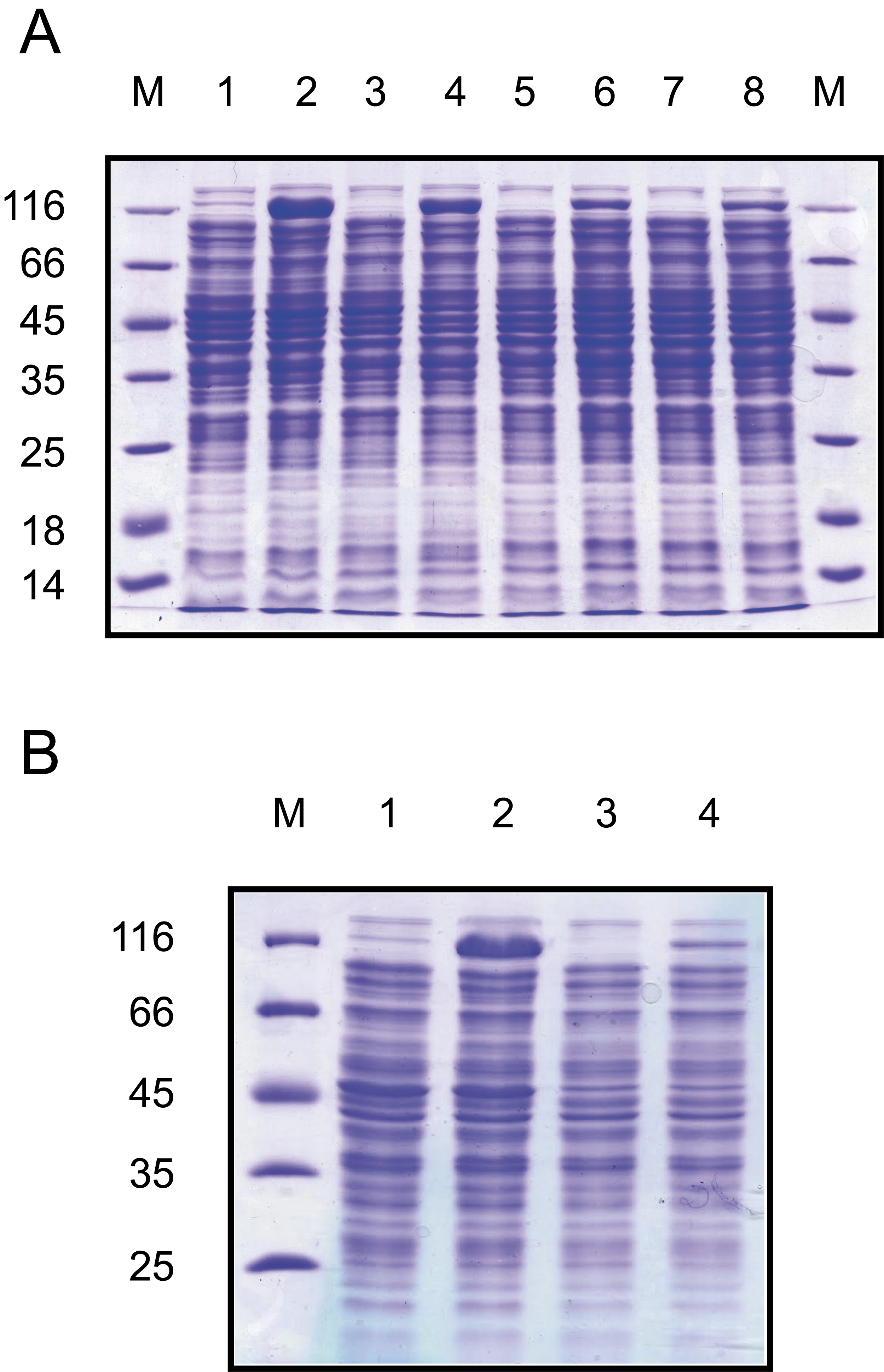

Supplement: S9 Fig — A SDS-PAGE of total proteins extracted from E. coli TOP10 overexpressing wt α subunit, or the D201A, H12A, and D19A variants (lanes 2, 4, 6, and 8, respectively). The corresponding protein extracts isolated from E. coli cultures not subjected to overexpression are reported in lanes 1, 3, 5, and 7. B SDS-PAGE of soluble proteins extracted from E. coli TOP10 overexpressing wild-type α subunit, or the D201A variant (lanes 2 and 4, respectively). The corresponding protein extracts isolated from E. coli cultures not subjected to overexpression are reported in lanes 1 and 3. (TIF) [file pone.0152915.s009.tif]

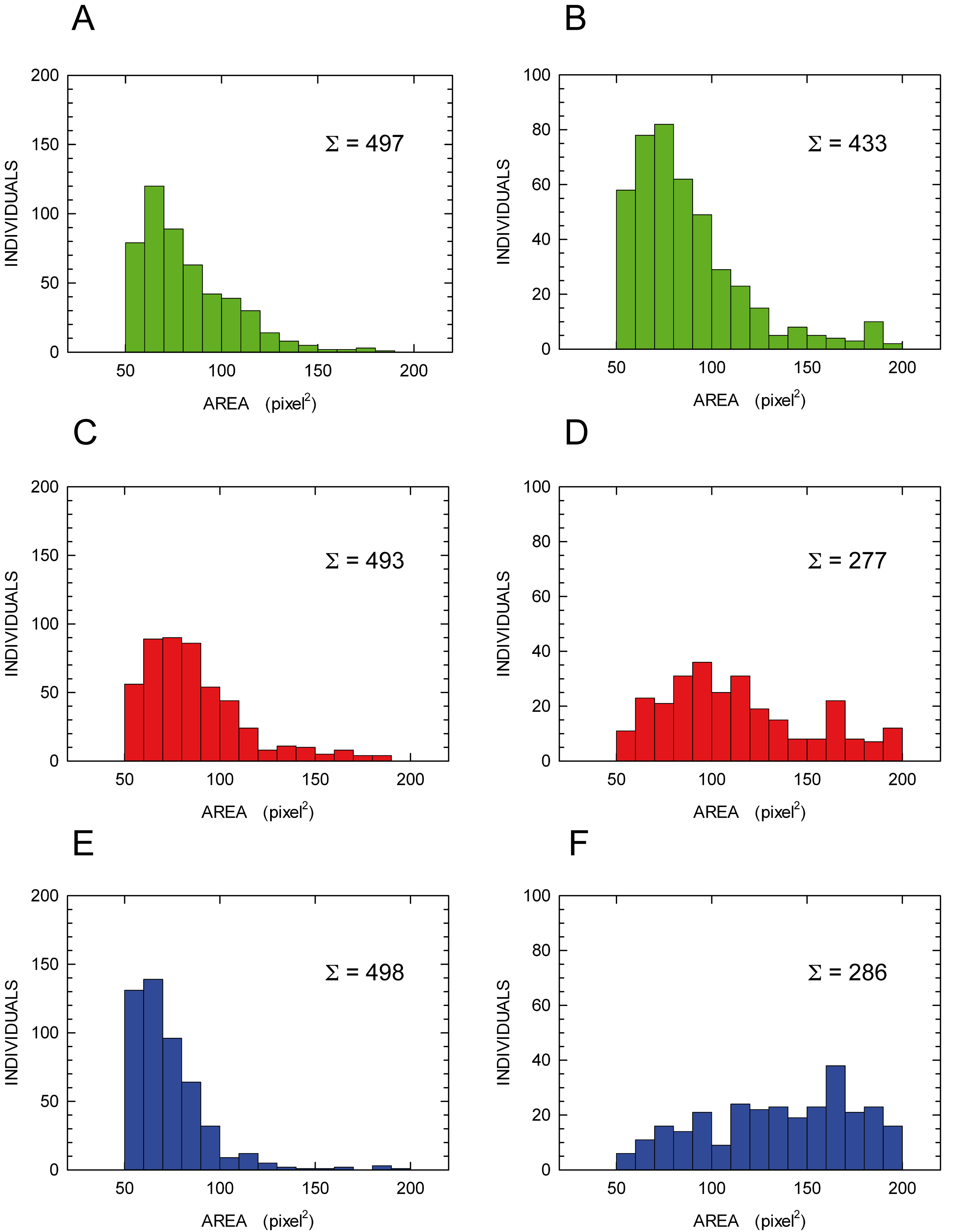

Supplement: S10 Fig — Size distribution of E. coli populations not induced (A,C,E) or induced (B,D,F) to overexpress wt α subunit (A,B), the D201A (C,D) or the H12A (E,F) variant. Bright-field micrographs of the different samples were acquired with a Nikon Eclipse 600 microscope. The images accordingly obtained were processed with the ImageJ software and, upon their conversion in binary format, cells area was determined. For every population 500 individuals were considered, and the sum of cells featuring area < 200 pixel2 is indicated in each panel. (TIF) [file pone.0152915.s010.tif]
